# Parallel metabolomic and transcriptomic profiling reveals the cytotoxic mechanism of protein-bound uremic toxin p-cresol sulfate via disruption of glutathione and glycerophospholipid metabolism in MC3T3-E1 osteoblasts

**DOI:** 10.1186/s12882-026-05004-x

**Published:** 2026-04-28

**Authors:** Qiong Wu, Jinhua Lan, Zhipeng Wan, Yu Liang, Kai Nie, Jielong Zhou, Bo Ji, Qiang Zhang, Zhihui Jiang, Xuefeng Huang, ZhiGuo Pan, Yu Shao

**Affiliations:** 1 School of Pharmacy, Southern Medical University, Guangzhou, 510515 China; 2 Department of Pharmacy, General Hospital of Southern Theater Command, Guangzhou, 510010 China; 3 Department of Traditional Chinese Medicine, General Hospital of Southern Theater Command, Guangzhou, 510010 China; 4 Department of Orthopaedics Oncology, Guangdong Provincial People’s Hospital (Guangdong Academy of Medical Sciences), Southern Medical University, Guangzhou, 510010 China; 5 School of Pharmacy, Guangzhou University of Chinese Medicine, Guangzhou, 510405 China; 6 The First School of Clinical Medicine, Southern Medical University, Guangzhou, 510515 China; 7 The First School of Clinical Medicine, Guangzhou University of Chinese Medicine, Guangzhou, 510405 China; 8 Department of Emergency, General Hospital of Southern Theater Command, Guangzhou, 510010 China; 9 Department of General Medicine, General Hospital of Southern Theater Command, Guangzhou, 510010 China

**Keywords:** Chronic kidney disease-mineral bone disorder, p-cresol sulfate, Comparative multi-omics analysis, Glutathione metabolism, Glycerophospholipid metabolism

## Abstract

**Background:**

Chronic kidney disease-mineral bone disorder (CKD-MBD), a common complication of chronic kidney disease (CKD), leads to vascular calcification, osteoporosis, and electrolyte disturbances, impacting patient survival and quality of life. Conventional dialysis poorly removes protein-bound uremic toxins like p-cresyl sulfate (PCS), which are linked to CKD-MBD. This study combined metabolomics and transcriptomics to explore PCS’s cytotoxic mechanisms in CKD-MBD for better clinical management.

**Methods:**

Using MC3T3-E1 osteoblasts treated with or without PCS, metabolic changes were analyzed via ultra-high performance liquid chromatography and quadrupole time-of-flight mass spectrometry (UHPLC-QTOF/MS), and transcriptomic changes through RNA sequencing (RNA-seq).

**Results:**

UHPLC-QTOF/MS identified 74 significantly altered metabolites and 15 disrupted metabolic pathways in PCS-treated osteoblasts. RNA-seq revealed 3,679 differentially expressed genes, with pathway analysis indicating significant disruptions in glutathione and glycerophospholipid metabolism, and alterations in cell apoptosis, cell cycle, and DNA repair processes.

**Conclusions:**

Parallel metabolomics and transcriptomics profiling showed PCS-induced disruptions in glutathione and glycerophospholipid pathways are central to cellular metabolic dysfunction in CKD-MBD. These insights highlight the potential of multi-omics to elucidate uremic toxin pathophysiology, providing a foundation for improved CKD-MBD management.

**Supplementary Information:**

The online version contains supplementary material available at 10.1186/s12882-026-05004-x.

## Introduction

Chronic kidney disease (CKD) is characterized by the systemic accumulation of uremic toxins (UTs) due to impaired renal excretion. These toxins not only exacerbate renal deterioration but also drive the progression of life-threatening complications, significantly reducing patient survival and quality of life [1]. UTs are classified into three categories based on molecular weight and protein-binding capacity [2]: (i) small water-soluble solutes (e.g., urea, creatinine), (ii) protein-bound solutes (e.g., p-cresol sulfate [PCS], indoxyl sulfate [IS]), and (iii) middle-molecular-weight compounds (e.g., parathyroid hormone, β2-microglobulin). While conventional hemodialysis effectively removes small solutes and modified techniques address middle molecules, protein-bound UTs remain largely unremovable due to their high albumin affinity [3]. Emerging evidence underscores that protein-bound UTs, particularly PCS, are pivotal contributors to multi-organ dysfunction in CKD, necessitating mechanistic investigations to elucidate their pathophysiological roles [4].

PCS, a gut microbiota-derived metabolite of aromatic amino acids, exemplifies the systemic toxicity of protein-bound UTs [5]. It induces endothelial dysfunction via leukocyte activation and oxidative stress, promotes cardiac apoptosis through Rho kinase and NADPH oxidase pathway, and exacerbates the progression of kidney disease by dysregulating the renin-angiotensin-aldosterone system and epithelial-mesenchymal transition [6–10]. Chronic kidney disease-mineral bone disorder (CKD-MBD) is a common complication of CKD. In addition to skeletal abnormalities, CKD-MBD can lead to anemia, pruritus, hypertension, heart failure, pericarditis, atherosclerosis, gastrointestinal bleeding, and other functional impairments across multiple systems, thereby significantly compromising patients’ quality of life [11, 12]. Notably, recent studies have highlighted the association between uremic toxins (UTs) and CKD-MBD [13, 14], although the underlying molecular mechanisms remain poorly understood. Moreover, there is currently limited research elucidating the specific mechanisms by which PCS contributes to CKD-MBD, underscoring the need for further investigation into the relationship between these two factors.

Parallel multi-omics approaches demonstrates remarkable advantages in the identification and screening of key metabolite molecules. Metabolomics, a cornerstone of systems biology, captures dynamic metabolic perturbations in response to pathological stimuli, offering direct insights into disease mechanisms [15, 16]. Coupled with transcriptomics, which profiles genome-wide RNA expression patterns [17], multi-omics integration enables holistic mapping of molecular networks from gene regulation to functional metabolite shifts [18, 19]. While ultra-performance liquid chromatography quadrupole time-of-flight mass spectrometry (UPLC-QTOF/MS) dominates metabolomic profiling due to its sensitivity and reproducibility, RNA-seq provides unparalleled resolution in transcriptome analysis. By combining metabolomics and transcriptomics data, potential target metabolites in cancer patients can be systematically identified and effectively screened [20–22]. Together, these approaches synergistically identify dysregulated pathways driving disease phenotypes [23].

In this study, we employ a comparative metabolomics-transcriptomics strategy to dissect PCS-induced cellular toxicity in MC3T3-E1 osteoblasts. Our preliminary data reveal that PCS disrupts glutathione and glycerophospholipid metabolism, impairing osteoblast viability and proliferation. These findings unveil metabolic reprogramming as a novel mechanism underlying CKD-MBD, providing a framework for targeting protein-bound UTs in therapeutic interventions.

## Materials and methods

### Cell culture

MC3T3-E1 osteoblasts (Center for Excellence in Molecular Cell Science, CAS, Shanghai, China) were cultured in a medium consisting of 90% MEMα supplemented with double antibiotics (NaHCO₃ 1.5 g/L, inositol 43.2 mg/L, folic acid 8.82 mg/L, β-mercaptoethanol 7.8 mg/L) and 10% high-quality fetal bovine serum (Gibco, Thermo Fisher Scientific, Waltham, MA, USA). The cells were maintained under standard conditions of 37 °C and 5% CO₂. Upon reaching 90% confluence, the cells were passaged. PCS was dissolved in DMSO and subsequently diluted to the desired concentration using the culture medium for treatment of the osteoblasts. The final concentration of DMSO in all media was maintained at or below 0.1% (v/v) [24]. The cells were then incubated for an additional period to allow further growth before analysis. Concurrently, the control group cells were treated with an equivalent volume of DMSO as a vehicle control to ensure that any observed effects were attributable to PCS rather than the solvent.

### Cell viability analysis

Logarithmic-phase cells were harvested, and 100 µL of cell suspension (approximately 1 × 10^5 cells per well) was added to each well of a 96-well culture plate. The cells were allowed to adhere and grow until they reached approximately 70–80% confluence. PCS was subsequently added to achieve final concentrations of 50 µg/mL, 100 µg/mL, 200 µg/mL, 500 µg/mL, and 1 mg/mL, and the cells were cultured for 24–48 h under standard conditions (37 °C, 5% CO_2_). Afterward, 10 µL of CCK-8 reagent (Beyotime Biotech Inc, Shanghai, China) was added to each well, followed by incubation for an additional 2 h. Finally, the optical density (OD) values at 450 nm were measured using an automated microplate reader.

### Cell apoptosis assay

Cell apoptosis was assessed using the Annexin-V/PI method [25]. Initially, cells were seeded in 6-well plates at a density of approximately 1 × 10^5 cells per well. Subsequently, cells were treated with PCS at concentrations of 100 µg/mL, 200 µg/mL, and 500 µg/mL and cultured for 48 h in an incubator under standard conditions (37 °C, 5% CO_2_). After trypsinization, MC3T3-E1 cells were collected by centrifugation at 2000 rpm for 5 min, and the supernatant was discarded. The cell pellet was washed three times with cold PBS and re-centrifuged under the same conditions. Cells were then resuspended in 300 µL of cold PBS to achieve a final concentration of approximately 3 × 10^6 cells/mL. For apoptosis analysis, 5 µL of Annexin-V/PI staining solution (Jiangsu KeyGEN BioTECH Corp., Ltd., Nanjing, China) was added to each sample, mixed gently, and incubated at room temperature in the dark for 15 min. Flow cytometry was performed using a flow cytometer (Beckman Coulter, Inc., USA) within 1 h to quantify apoptotic cells. Data were analyzed with FlowJo v10.9.0 software.

### Cell cycle determination

To investigate the effects of PCS on the cell cycle of osteoblasts, we analyzed the cell cycle distribution of MC3T3-E1 osteoblasts. The drug treatment and culture procedures were identical to those in the apoptosis experiment. Briefly, cells were harvested, the culture medium was removed, and the cells were washed with PBS. Subsequently, the cells were fixed with pre-cooled 75% ethanol for 4–6 h in the dark for membrane permeabilization. After centrifugation to discard the ethanol, 300 µL of PI staining solution was added to each sample and incubated in the dark at room temperature for 30 min. Finally, the cell cycle distribution was determined using a flow cytometer (Beckman Coulter, Inc., USA), and data were analyzed with FlowJo v10.9.0 software.

### Cell experiments

5 × 10^6 cells/mL were seeded into 10-cm cell culture dishes. PCS was added to achieve a final concentration of 500 µg/mL, with the final DMSO concentration maintained at or below 0.1% (v/v). An equal volume of DMSO (vehicle control) was added to the blank control group. For transcriptomics analysis, three independent biological replicates per group were used. For metabolomics analysis, three independent biological replicates per group were used.The 500 µg/mL concentration was selected for subsequent omics analysis as it represented the minimal effective dose inducing significant phenotypes, thereby providing a window to investigate specific pathological mechanisms prior to overwhelming cytotoxicity.

### Metabolomics analysis

#### Sample processing

The culture medium was removed from the cell culture dish, and the cell surface was promptly rinsed with an appropriate volume of PBS buffer. Thereafter 1 mL of pre-cooled ice-cold methanol was added to quench the cells, which were then placed in a -80 °C freezer for 15 min to ensure complete quenching. Cells were scraped off the surface of the culture dish using a cell scraper and transferred to a microcentrifuge tube. After centrifugation at 13,000 × g for 15 min, the supernatant was collected, dried under nitrogen, and resuspended in 100 µL of methanol: acetonitrile (volume ratio 3:7). Following another centrifugation at 13,000 × g for 10 min, 75 µL of the supernatant was transferred to an LC-MS injection vial for subsequent analysis. Additionally, 5 µL of each sample was pooled to prepare quality control (QC) samples.

#### Chromatographic conditions

Reverse-phase chromatography was performed using an Agilent 1290 Infinity liquid chromatography system equipped with an Acqµity UPLC BEH C18 column (2.1 mm × 100 mm, 1.7 μm). The mobile phase consisted of A (water containing 0.1% formic acid) and B (acetonitrile containing 0.1% formic acid). Gradient elution was employed with the following program: 0–2 min, 5% B; 2–20 min, 5%–95% B, followed by a 5-min equilibration period. The flow rate was set at 0.35 mL/min, and the injection volume was 4 µL.

#### Mass spectrometry conditions

Mass spectrometric analysis was conducted on an Agilent 6530 Accurate-Mass Quadrupole-Time of Flight Mass Spectrometer using electrospray ionization (ESI) in both positive and negative ion modes. The parameters were as follows: capillary voltage, 3500 V; drying gas flow rate, 11 L/min; drying gas temperature, 350 °C; nebulizer pressure, 45 psig; collision voltage, 120 V; skimmer voltage, 60 V. Data acquisition was performed over a mass-to-charge ratio range of 50–1000. Two ions with mass-to-charge ratios of 121.0509 and 922.0098 were used as internal standards for real-time mass correction.

#### Data processing

Raw LC-MS data were imported into Agilent MassHunter Qualitative Analysis software and exported in mzData format. Peak alignment and integration were performed using XCMS (http://metlin.scripps.edu/download), resulting in a three-dimensional data matrix comprising mass-to-charge ratios, retention times, and peak intensities. XCMS parameters were set to default values except for fwhm = 10, bw = 10, and snthresh = 5. Missing values were removed according to the “80% rule,” retaining only metabolites detected in more than 80% of the samples for further analysis [26]. The mass spectrometry response was normalized based on the number of cells and total peak area per sample. Normalized data were subsequently imported into Simca-P v11.0 (Umetrics, Sweden) for multivariate statistical analysis. First, principal component analysis (PCA) was performed for unsupervised data overview. Then, partial least squares-discriminant analysis (PLS-DA) was applied to maximize group separation. The PLS-DA model was validated by 200 permutation tests to guard against overfitting. Differential metabolites were identified based on a variable importance in projection (VIP) score > 1.0, combined with an independent sample t-test (*p* < 0.05) and a fold change > 1.5. Differential features were putatively identified by matching their accurate mass (mass error < 5 ppm) against the Human Metabolome Database (HMDB). According to the Metabolomics Standards Initiative (MSI) guidelines, these metabolites are designated as Level 3 (putatively characterized compound classes). The identification confidence level for each metabolite is provided in Supplementary Table S1. To elucidate the significantly altered metabolic pathways in osteoblasts after PCS treatment, the identified differential metabolites and their relative abundances were uploaded to the MetaboAnalyst platform (http://www.metaboanalyst.ca). Pathway analysis was performed using MetPA (http://metpa.metabolomics.ca/), which integrates prior knowledge-based pathway enrichment and topology analyses [27]. The software matched the uploaded metabolite IDs against its database (HMDB ID, PubChem ID, KEGG ID) and cross-referenced them with the mouse metabolic pathway database. Ultimately, the most significantly altered metabolic pathways in MC3T3-E1 osteoblasts following PCS treatment were identified.

### Transcriptome analysis

#### Total RNA extraction and quality control

When the cell density reached 5 × 10^6 cells per dish, the culture medium was carefully aspirated, and the cells were washed three times with PBS buffer. The dish was then tilted slightly to remove residual PBS, followed by the addition of 1 mL of Trizol lysis buffer (Gibco, Thermo Fisher Scientific, Waltham, MA, USA). The dish was placed flat to ensure the lysis buffer fully covered the cells, and the cells were lysed by pipetting up and down 8–10 times until complete detachment. The lysate was immediately transferred into a cryotube and stored at -80 °C. Total RNA concentration was measured using an Agilent 2200 nucleic acid analyzer (Agilent, USA). Only RNA samples with an A260/A280 ratio between 1.8 and 2.2, an RNA Integrity Number (RIN) ≥ 7.0, and a minimum concentration of 50 ng/µL were considered qualified and used for subsequent transcriptome sequencing. All subsequent transcriptome sequencing experiments were conducted by Shanghai Jiyan Biotechnology Co., Ltd.

#### Library construction and sequencing

Poly(A) RNA was isolated following the instructions of the Dynabeads^®^ mRNA Purification Kit. Ten microliters of poly(A) RNA solution was added to a 0.2 mL centrifuge tube, followed by the addition of 1 µL of 10× RNase III Reaction Buffer and 1 µL of RNase III. The mixture was gently mixed by pipetting five times and briefly centrifuged. After incubation at 37 °C for 3 min, 20 µL of Nuclease-Free water was added, and the tube was placed on ice to fragment the RNA. Next, 5 µL of Nucleic Acid Binding Beads, 90 µL of Binding Solution Concentrate, and 30 µL of the digested product were added to a 1.5 mL centrifuge tube, followed by 150 µL of 100% ethanol. The mixture was vortexed and incubated at room temperature for 5 min. The tube was placed on a magnetic stand for 5–6 min, and the supernatant was discarded. The beads were washed twice with 150 µL of Wash Solution Concentrate, dried at room temperature for 1–2 min, and eluted into a new centrifuge tube. The purified RNA fragments were obtained. Adapters were ligated to the RNA, and reverse transcription was performed according to the Ion Total RNA-Seq Kit v2 protocol. The resulting cDNA was purified, PCR-amplified, and re-purified to construct the sequencing library. Library quality was assessed using the Agilent 2200 nucleic acid analyzer. Quality control steps included library dilution (100 pM) and droplet PCR on the Ion OneTouch™ 2 system. Sequencing was performed after library preparation, including instrument cleaning (chlorine wash, water wash), initialization, chip loading, and RNA-seq data collection.

#### Transcriptome data analysis

RNA-seq was performed on three biological replicates per group. Raw RNA-seq data from the blank control and PCS treatment groups were filtered to remove adapter and contaminant sequences. Clean reads were mapped to the reference genome (mm10) using HISAT2, and mapped reads were quantified for gene expression. RPKM values were calculated for visualization purposes only. Differential expression analysis was performed based on Student’s t-test using R software (version 3.6.0). Genes with an absolute |log2 fold change| > 1 and a false discovery rate (FDR) < 0.05 (Benjamini-Hochberg correction) were considered significantly differentially expressed. To account for differences in library size, raw counts were normalized using the TMM method implemented in the edgeR package prior to statistical analysis.Functional enrichment analysis (GO analysis) was performed on the differentially expressed genes using databases such as NCBI, Swissprot/Uniprot, The Gene Ontology, and AmiGo. GO terms were annotated and classified, and significance was evaluated based on discrete distribution, FDR, and enrichment analysis. This revealed the biological functions affected by PCS stimulation in osteoblasts. Additionally, pathway enrichment analysis (KEGG pathway analysis) was conducted on the differentially expressed genes using integrated databases such as NCBI, KEGG, Biocarta, and Reactome. Pathways were classified based on KEGG annotations, and significance was determined through discrete distribution analysis. This identified the pathways, interacting proteins, cellular functions, and metabolic processes altered by PCS treatment in osteoblasts.

### Comparative analysis of disease-related differential metabolites and expressed genes functionality

To comprehensively identify the cellular biological functions significantly altered after PCS treatment at both metabolic and transcriptional levels, and to better understand the toxic effects of PCS on osteoblast cellular function by combining upstream gene regulation and downstream functional molecular responses, we performed an enrichment analysis of diseases and functions using the IPA (Ingenuity Pathway Analysis) software from QIAGEN’s cloud-based data analysis platform (Ingenuity, Redwood City, CA, USA). This analysis was conducted on the differential metabolites identified through metabolic profiling and the differentially expressed genes obtained via transcriptome sequencing. The results were ranked according to their scores. In this study, we did not consider the direct effects of one molecule on another or the fold changes of molecules in the dataset but focused solely on the correlation between the imported data and known functions or biological processes that are not attributable to random non-matching. Therefore, the right-sided Fisher’s Exact Test was applied to calculate p-values, with *p* < 0.05 indicating negligible chances of random matching. Additionally, the Z-score was used to assess the predicted outcomes of upstream or downstream processes, as well as the interaction effects between molecules or the impact of fold changes in the dataset on biological processes. Typically, a Z-score > 2 indicates significant activation of the corresponding molecule/function, while a Z-score < 2 indicates significant inhibition. Overall, a p-value < 0.05 combined with a larger absolute Z-score value reflects more pronounced changes in cellular functions.

## Results

### PCS suppressed the viability of MC3T3-E1 cells in a dose-dependent manner

**Fig. 1:**
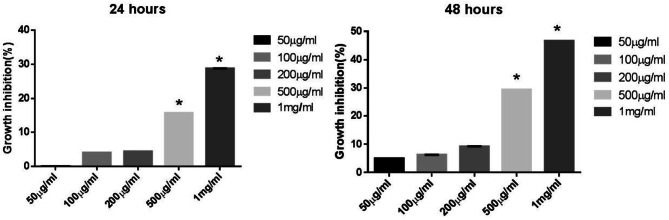
Cell viability evaluated by CCK-8 assay. MC3T3-E1 cells were seeded in 96-well plate and treated with 50 µg/mL, 100 µg/mL, 200 µg/mL, 500 µg/mL, 1 mg/mL PCS for 24 h and 48 h. The growth inhibition of PCS on MC3T3-E1 was evalµated based on the absorbance at 450 nm. All the measurements were carried out in five replicates, *p < 0.05,compared with control group

MC3T3-E1 cells were treated with PCS for 24 h and 48 h at concentrations of 0 µg/mL, 50 µg/mL, 100 µg/mL, 200 µg/mL, 500 µg/mL, and 1 mg/mL. The cell viablity of all groups were measured using the CCK-8 assay, follow by cell growth inhibition calculation based on 0 µg/mL control group,. The results demonstrated that PCS significantly inhibited the proliferation of MC3T3-E1 osteoblasts when the concentration reached 500 µg/mL. In contrast, no significant differences in growth inhibition were observed among the treatment groups exposed to 50 µg/mL, 100 µg/mL, and 200 µg/mL PCS compared with the blank control group, suggesting that there was almost no inhibitory effects of PCS on osteoblast proliferation when the concentration below 200 µg/mL. For detailed information, please refer to Fig. 1.

### PCS induced MC3T3-E1 cells into apoptosis status

**Fig. 2:**
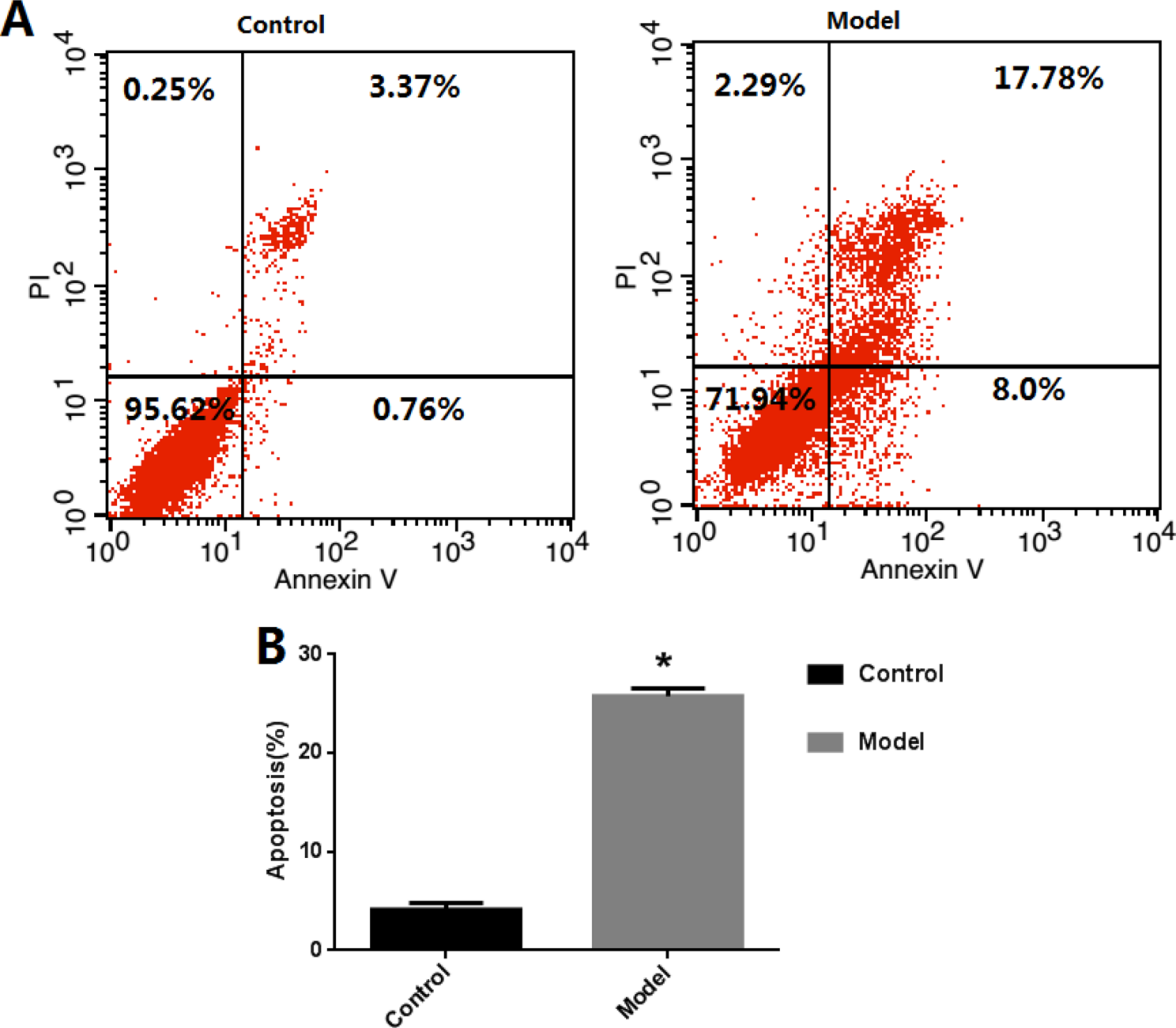
PCS (500 µg/mL) induces apoptosis in MC3T3-E1 cells. (**A**) Impact of PCS on apoptosis of MC3T3-E1 cells. The percentage of annexin-stained apoptotic cells was analyzed by cytometry and is summarized in a histogram. (**B**) Quantification of apoptotic cells (%). The control group (cells without PCS treatment) is shown. All the measurements were carried out in triplicates, *p < 0.05, compared with control groups

MC3T3-E1 cells were treated with PCS at concentrations of 50 µg/mL, 100 µg/mL, 200 µg/mL, 500 µg/mL, and 1 mg/mL, followed by incubation in a cell culture incubator for 24 h and 48 h. The pro-apoptotic effects of different PCS concentrations on osteoblasts were evaluated using the Annexin-V/PI method. The results demonstrated that when the PCS concentration reached 500 µg/mL and acted for 48 h, it significantly promoted apoptosis in MC3T3-E1 osteoblasts, with an apoptosis rate of approximately 25.78%, which was about 20% higher than the normal apoptosis rate. In contrast, no significant differences in apoptosis rates were observed between the groups treated with 50 µg/mL, 100 µg/mL, and 200 µg/mL of PCS and the blank control group. For detailed information, please refer to Fig. 2.

### PCS promoted the cell cycle of MC3T3-E1 cells arrest in G0/G1 phase

**Fig. 3:**
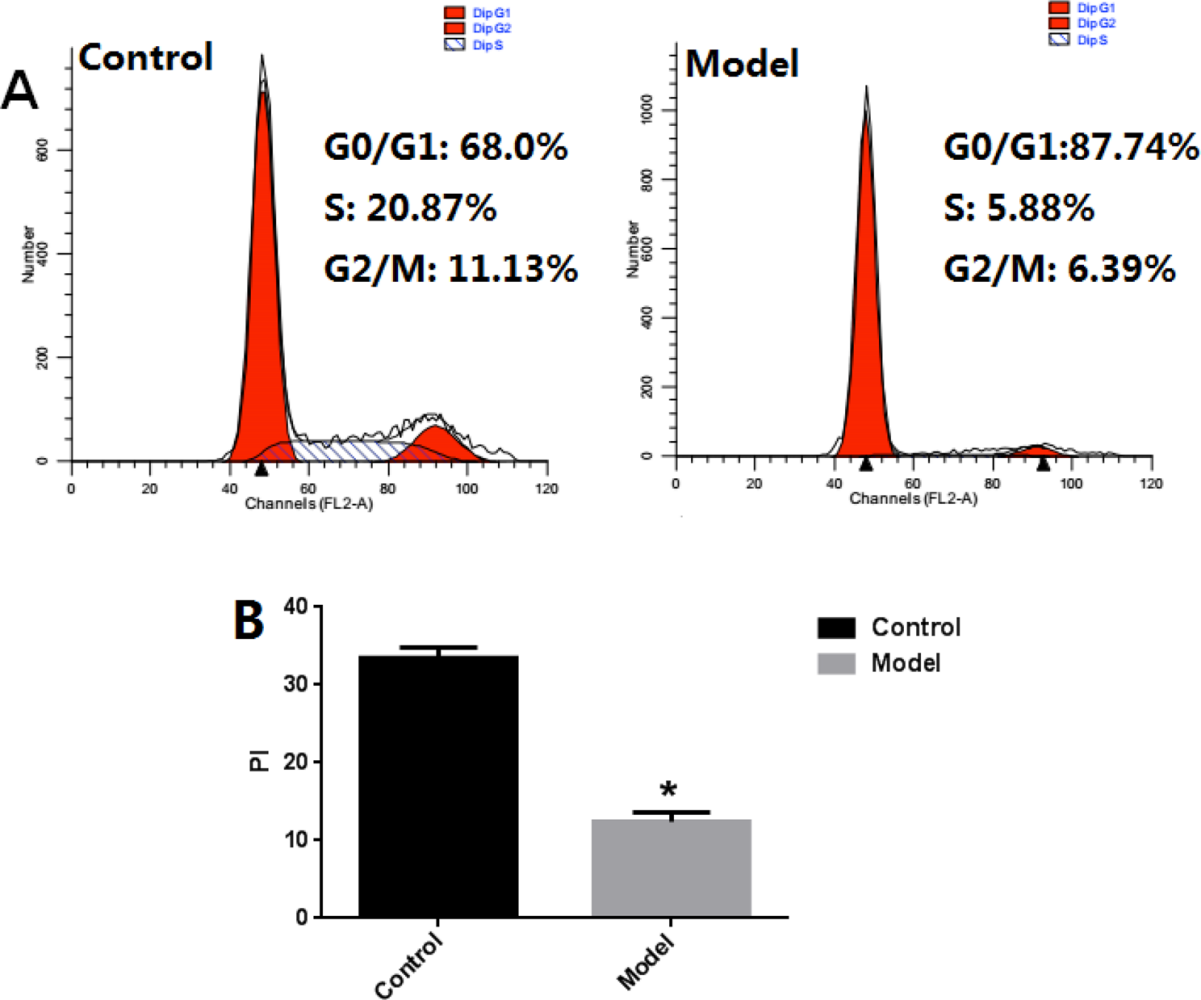
(**A**) Impact of PCS on cell cycle of MC3T3-E1 cells. PI was calculated by the ratio of number of cells in S phase and G2/M phase to number of cells in the whole cell cycle, and is summarized in a histogram. (**B**) Nature cell cycle was grouped as control, and all the measurements were carried out in triplicates, *p < 0.05, compared with control groµps

To determine the optimal PCS concentration for subsequent omics experiments, we investigated the changes in the cell cycle of MC3T3-E1 cells upon PCS treatment. The proliferation index (PI), defined as the proportion of cells in the S and G2/M phases relative to the total cell population, was used as a measurement standard to evaluate the proliferation rate of the studied cells. The results demonstrated that when the PCS concentration reached 500 µg/mL after 48 h of exposure, it significantly disrupted the normal cell cycle of MC3T3-E1 osteoblasts, reducing the PI to approximately 12.27%, which is about 20% lower than that of untreated control cells. This suggests that following PCS treatment, a greater proportion of cells were arrested in the G0/G1 phase, indicating that the proliferation process of MC3T3-E1 cells was markedly inhibited by PCS. For further details, refer to Fig. 3.

### Metabolomics results

#### Metabolic profiles of cell extracts

**Fig. 4:**
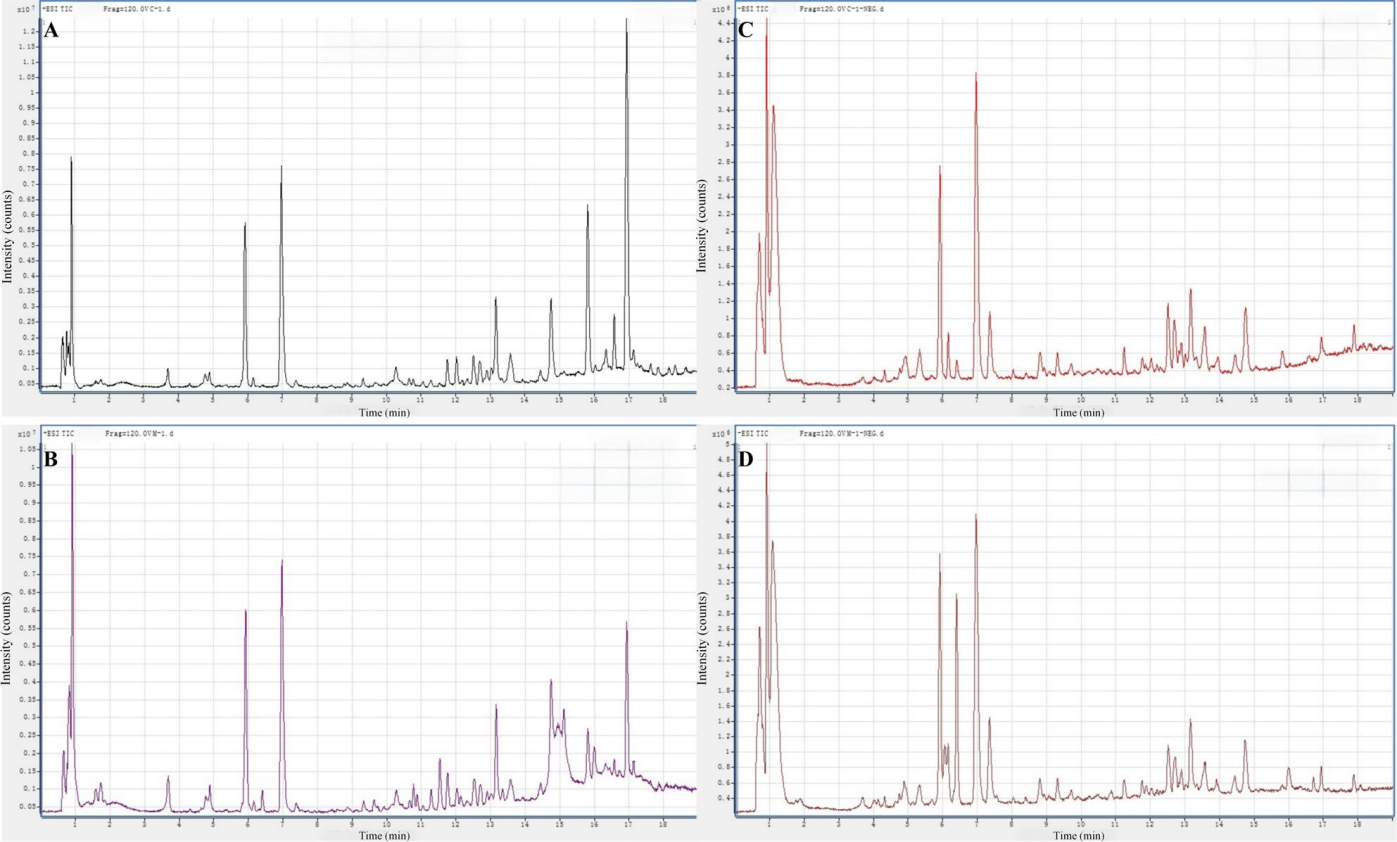
The typical total current chromatograms (TICs) of intracellular extract separated on LC-MS. (**A**) control group in positive mode. (**B**) PCS treated group in positive mode. (**C**) control group in negative mode. (**D**) PCS treated group in negative mode

Based on the results of the previous CCK-8 assay, apoptosis analysis, and cell cycle determination, MC3T3-E1 osteoblasts were treated with 500 µg/mL PCS for 48 h to establish the experimental model in this study. Intracellular extracts from both the control group and the PCS treatment group were collected and analyzed using UHPLC-QTOF/MS to investigate the intracellular metabolic changes in osteoblasts following PCS treatment. Figure 4 displays the typical total ion chromatograms (TICs) of the blank control group and the PCS treatment group in both positive and negative ion modes. After a series of data preprocessing steps, including peak alignment, normalization, and peak integration, 1169 ion peaks were identified in the positive ion mode and 581 ion peaks in the negative ion mode from the LC-MS dataset.

#### Multivariate statistical analysis and identification of intracellular metabolites

**Fig. 5:**
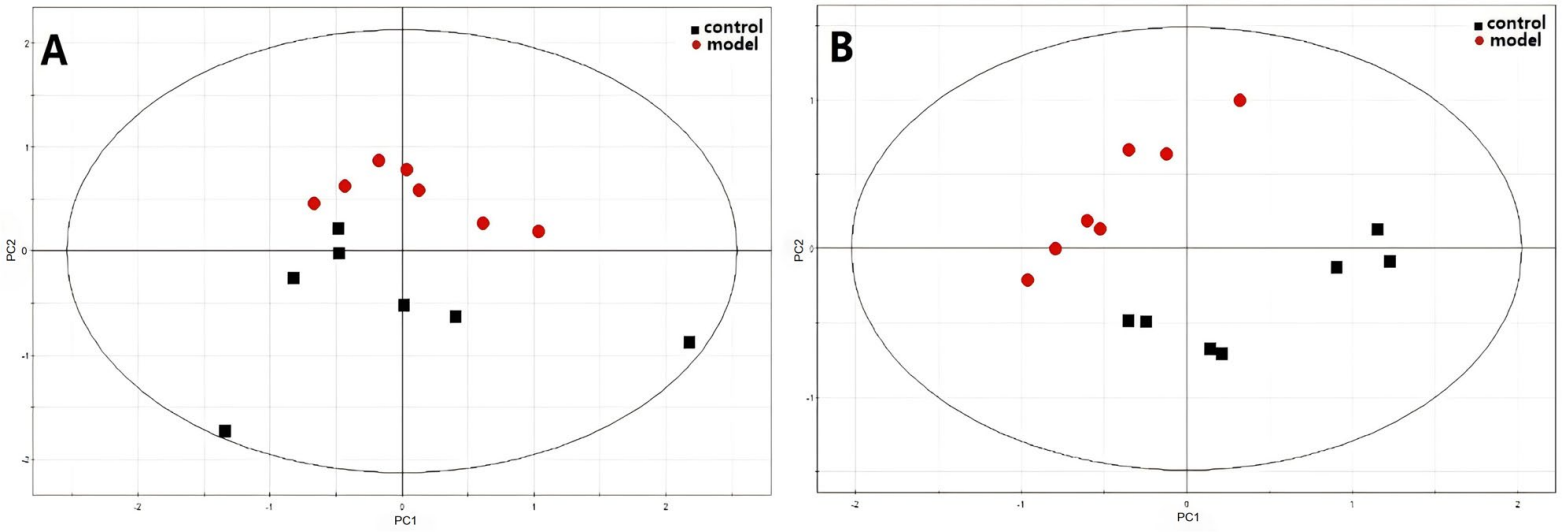
The PCA score plots of control group and model group (PCS treated). (**A**) in positive mode. (**B**) in negative mode

**Fig. 6:**
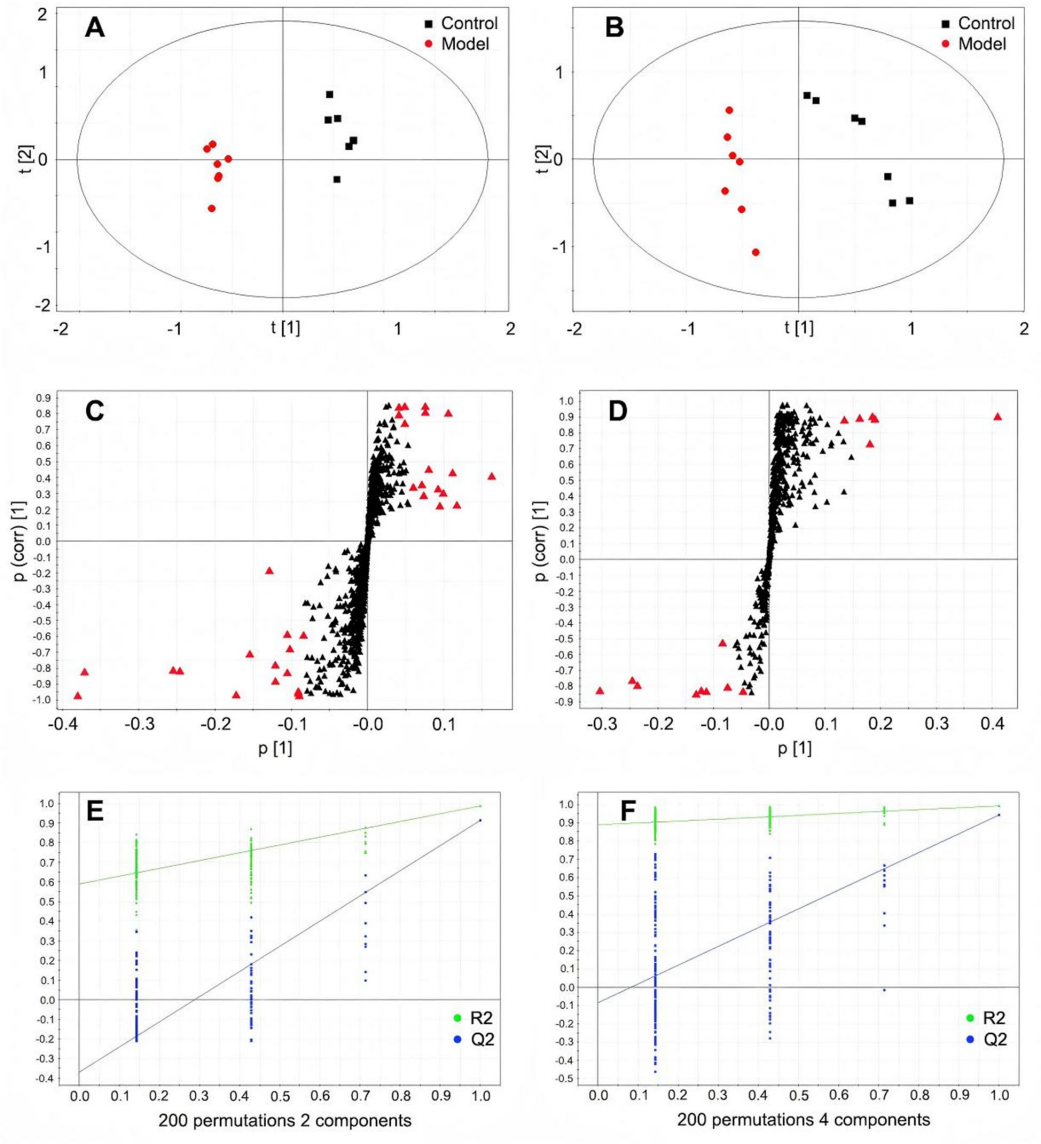
Multivariate analysis of metabolic profiles in MC3T3-E1 osteoblasts. (**A**, **B**) PLS-DA score plots for the control and PCS-treated groups in positive (**A**) and negative (**B**) ion modes. (**C**, **D**) Corresponding S-plots. (**E**, **F**) Validation plots from 200 permutation tests

After peak alignment, peak integration, and normalization of the LC-MS data, multivariate statistical analysis was applied to reveal the changes in the intracellular metabolome of MC3T3-E1 cells following PCS treatment. First, we employed PCA to visualize the separation and trends between the blank control group and the PCS-treated group. As shown in Fig. 5, under both positive and negative ion modes, the PCA score plots demonstrate a distinct separation trend between the blank control group and the PCS-treated group, indicating that the intracellular metabolome of MC3T3-E1 osteoblasts underwent significant alterations after PCS treatment. To identify these altered small metabolite variables, we subsequently applied a supervised pattern recognition method—partial least squares-discriminant analysis (PLS-DA)—for in-depth analysis of the LC-MS data. As illustrated in Fig. 6A and B, the PLS-DA score plots show clear clustering and separation between the blank control group and the PCS-treated group. In the positive ion mode, the PLS-DA model comprises four components with R² = 0.989 and Q²(cum) = 0.915. In the negative ion mode, the model comprises two principal components with R² = 0.993 and Q² (cum) = 0.942. To ensure that the constructed PLS-DA model is not overfitted, we performed 200 permutation tests for validation, as shown in Fig. 6E and F. In the positive ion mode, the intercepts for R² and Q² are 0.626 and − 0.548, respectively. In the negative ion mode, the intercepts for R² and Q² are 0.888 and − 0.0843, respectively. These results confirm that the PLS-DA model based on LC-MS data exhibits excellent predictive ability and robustness. Based on this reliable and robust model, differential variables between the blank control group and the PCS-treated group were screened using the constructed PLS-DA model. As depicted in Fig. 6C and D, variables located farther from the center point of the S-plot contribute more significantly to the separation trend between the two groups. According to the screening criteria of variable importance in projection (VIP) > 1, independent sample t-test (*p* < 0.05), and fold change > 1.5, a total of 74 differential variables were identified between the blank control group and the PCS-treated group. The detailed results are presented in Table S1.

#### Metabolic pathway analysis

**Fig. 7:**
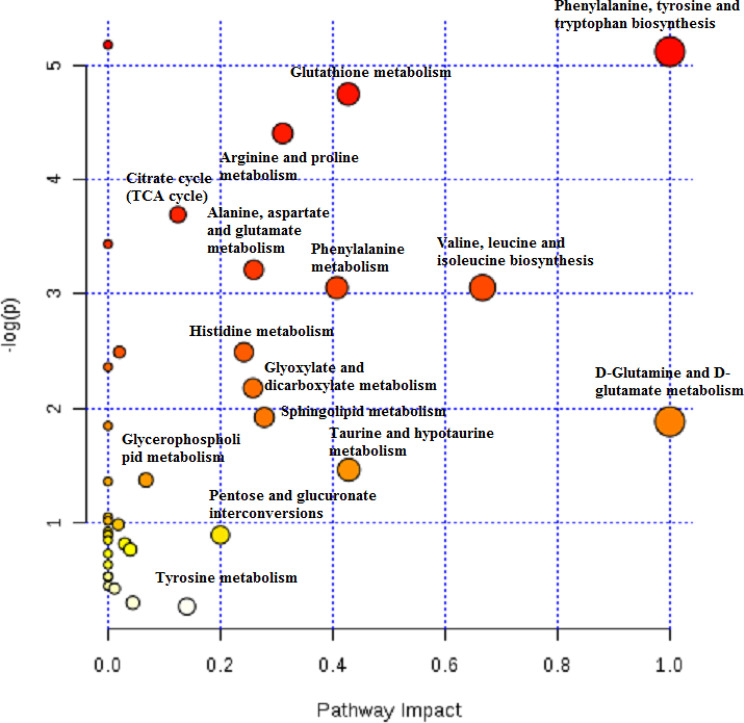
Summary of the fifteen important metabolic pathways (Phenylalanine, tyrosine and tryptophan biosynthesis, D-Glutamine and D-glutamate metabolism, Valine, leucine and isoleucine biosynthesis, Taurine and hypotaurine metabolism, Glutathione metabolism, Phenylalanine metabolism, Arginine and proline metabolism, Sphingolipid metabolism, Alanine, aspartate and glutamate metabolism, Glyoxylate and dicarboxylate metabolism, Histidine metabolism, Pentose and glucuronate interconversions, Citrate cycle (TCA cycle), Glycerophospholipid metabolism) identified by MetPA

The aim of metabolomics research is not only to identify changes in the intracellular metabolome of osteoblasts after PCS stimulation but also to uncover the most significantly altered metabolic pathways following PCS treatment. These significantly altered metabolic pathways are critical for elucidating the biological effects of PCS on osteoblasts. Therefore, in this study, the pathway analysis was performed using the MetPA module integrated within the MetaboAnalyst platform was employed to characterize the significantly altered metabolic pathways in osteoblasts after PCS treatment and further investigate the potential mechanisms underlying PCS-induced osteoblast damage. In this study, the importance of the node position of metabolic pathways was used as an evaluation criterion for pathway significance. In the topological analysis of metabolic pathways, we considered a metabolic pathway with an importance value greater than 0.05 as a potential target pathway. The specific results are presented in Fig. 7; Table 1. In Fig. 7, all metabolic pathways are represented by dots, where larger and darker dots indicate more important node positions within the metabolic network. The results demonstrated that 15 metabolic pathways were significantly altered after PCS stimulation of osteoblasts, including phenylalanine, tyrosine, and tryptophan biosynthesis; D-glutamine and D-glutamate metabolism; valine, leucine, and isoleucine biosynthesis; taurine and hypotaurine metabolism; glutathione metabolism; phenylalanine metabolism; arginine and proline metabolism; sphingolipid metabolism; alanine, aspartate, and glutamate metabolism; glyoxylate and dicarboxylate metabolism; histidine metabolism; pentose and glucuronate interconversion; tyrosine metabolism; citric acid cycle (TCA cycle); and glycerophospholipid metabolism.

**Table 1:** Results of pathway enrichment and topology analysis using MetPA^a^

No.	Pathway name	Total	Expected	Hits	Raw *p*	FDR	Impact
1	Phenylalanine, tyrosine and tryptophan biosynthesis	4	0.12985	2	5.94E-03	0.24	1.00
2	D-Glutamine and D-glutamate metabolism	5	0.16231	1	1.52E-01	0.81	1.00
3	Valine, leucine and isoleucine biosynthesis	11	0.35709	2	4.71E-02	0.43	0.67
4	Taurine and hypotaurine metabolism	8	0.2597	1	2.33E-01	1.00	0.43
5	Glutathione metabolism	26	0.84404	4	8.61E-03	0.24	0.43
6	Phenylalanine metabolism	11	0.35709	2	4.71E-02	0.43	0.41
7	Arginine and proline metabolism	44	1.4284	5	1.22E-02	0.25	0.31
8	Sphingolipid metabolism	21	0.68172	2	1.47E-01	0.81	0.28
9	Alanine, aspartate and glutamate metabolism	24	0.77911	3	4.02E-02	0.43	0.26
10	Glyoxylate and dicarboxylate metabolism	18	0.58433	2	1.14E-01	0.72	0.26
11	Histidine metabolism	15	0.48694	2	8.28E-02	0.62	0.24
12	Pentose and glucuronate interconversions	16	0.51941	1	4.12E-01	1.00	0.20
13	Tyrosine metabolism	44	1.4284	1	7.71E-01	1.00	0.14
14	Citrate cycle (TCA cycle)	20	0.64926	3	2.48E-02	0.41	0.12
15	Glycerophospholipid metabolism	30	0.97389	2	2.54E-01	1.00	0.07

^a^: Total is the total number of compounds in the pathways; the hits is the actually matched number from the user upload data; the raw p is the original p value calculated from the enrichment analysis; the impact is the pathway impact value calculated from pathway topology analysis

### Transcriptome sequencing results

#### RNA quality inspection results

**Fig. 8:**
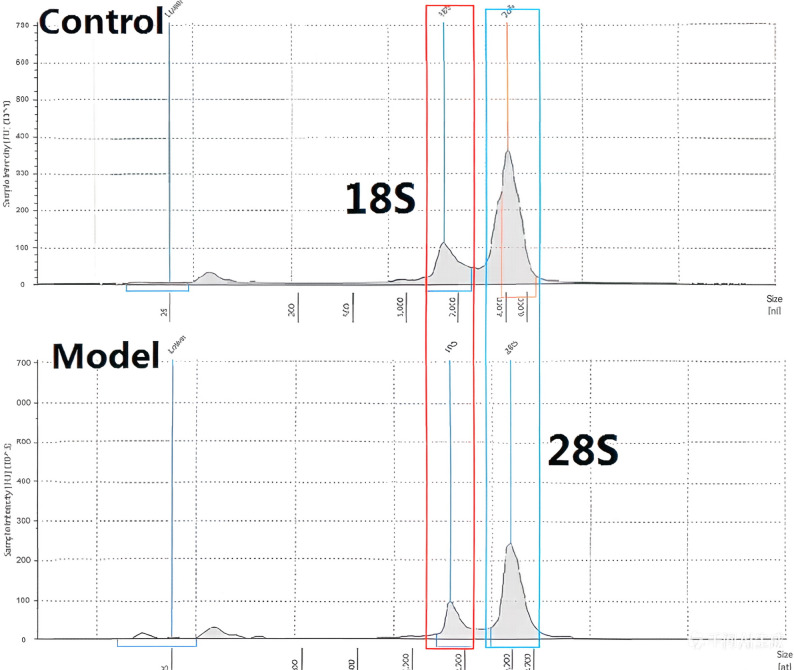
Representative capillary electrophoresis profiles of total RNA extracted from the blank control group (top) and the PCS-treated model group (bottom) using the Agilent 2200 Bioanalyzer

The total RNA concentration extracted from each sample was quantified using the Agilent 2200 Bioanalyzer (Agilent, USA). Only samples that passed the RNA quality inspection were eligible for subsequent transcriptome sequencing analysis. The RNA quality inspection revealed that all samples exhibited two distinct bands at 28 S and 18 S on the capillary electrophoresis profile, with no additional impurity bands observed. Furthermore, Fig. 8 present representative peak profiles of the blank control group and the PCS-treated group. As shown in the figures, the peaks at 28 S and 18 S are well-defined and free of impurity peaks. Additionally, the 28 S/18S ratios for all samples exceeded 1.5, confirming that the RNA was intact and not significantly degraded. These results indicate that all samples passed the quality inspection and are suitable for proceeding with transcriptome sequencing.

#### Evaluation of effective sequencing volume

**Fig. 9:**
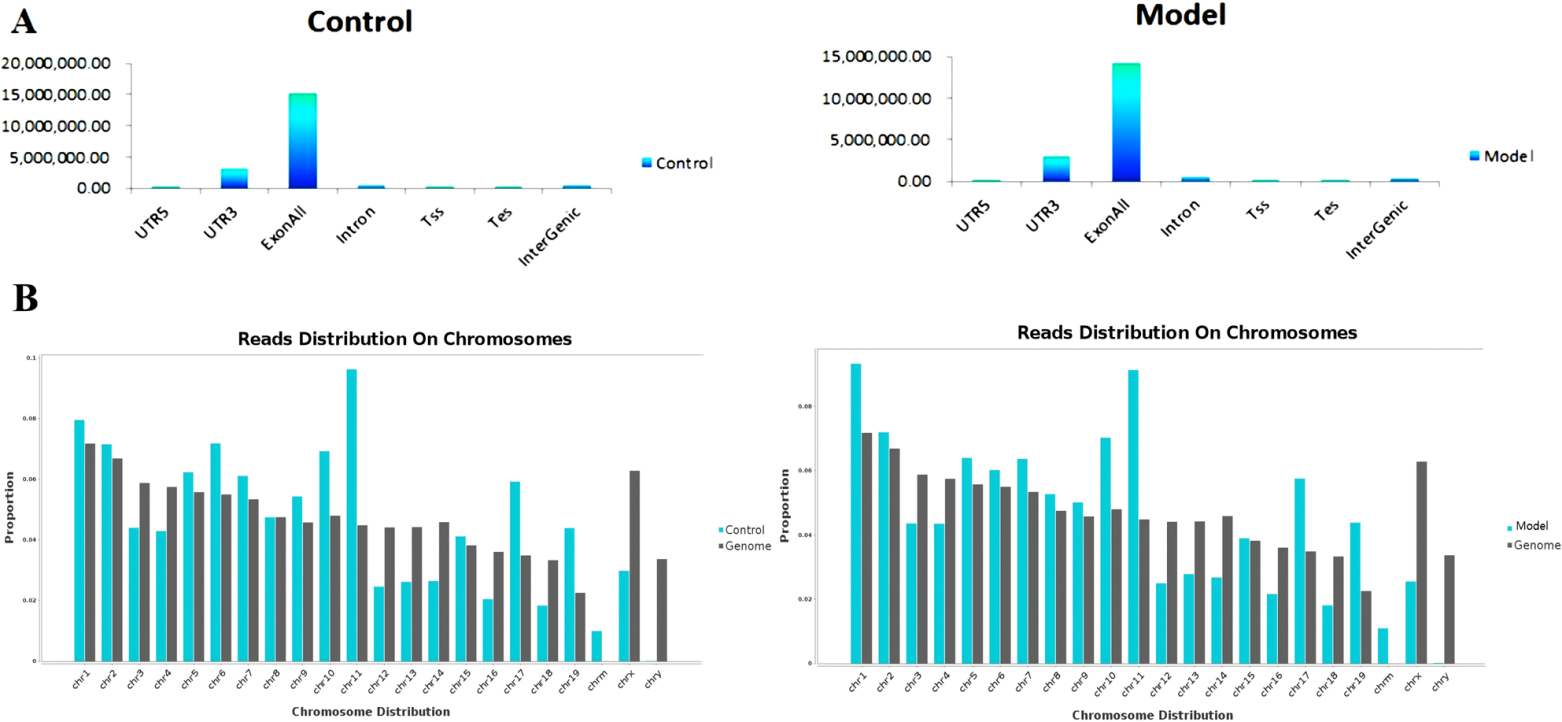
(**A**) Gene structure analysis: Bar graphs depicting the classification and quantitative distribution of mapped RNA-seq reads across different gene structural features, including 5’ untranslated region (UTR5), 3’ untranslated region (UTR3), exonic regions (ExonAll), introns, transcription start sites (Tss), transcription end sites (Tes), and intergenic regions. (**B**) Chromosomal Reads Distribution: Histograms showing the proportional distribution of mapped RNA-seq reads across the 21 pairs of chromosomes in both control and PCS-treated samples

The raw data from RNA-seq sequencing of the blank control group and the PCS-treated group were filtered to remove adapter sequences and contaminants. The resulting Clean Reads were subsequently mapped to the reference genome sequence, yielding Mapped Reads that represent the effective sequencing volume in the experiment. All samples exhibited high-quality sequencing data, with valid read percentages exceeding 95% (see Supplementary Table S2). In the mapping statistics of transcriptomic data, the matching rate (MappedRate%) was above 85% for all samples (Table 2). In the gene structure diagram, exons constituted the largest proportion, while introns accounted for a very small fraction (Fig. 9A). Additionally, RNA distribution across the 21 pairs of chromosomes was uniform in all samples, with no significant bias toward any single chromosome (Fig. 9B). These findings confirm that the RNA quality in all analyzed samples was satisfactory, free from contamination, and unlikely to produce false-negative results, thus enabling subsequent differential gene screening.

**Table 2:** Mapping statistics for transcriptome sequencing

Sample	All	UnMapped	Mapped	MappedRate%	UniqueMapped	UniqueMappedRate%
Control1	16,889,710	895,621	15,994,089	94.7	14,787,634	87.6
Control2	14,066,769	868,334	13,198,435	93.8	12,142,727	86.3
Control3	16,599,434	1,146,545	15,452,889	93.1	14,375,442	86.6
Model1	16,159,659	1,071,413	15,088,246	93.4	14,098,470	87.2
Model2	16,255,976	865,360	15,390,616	94.7	14,423,527	88.7
Model3	15,711,523	866,728	14,844,795	94.5	13,827,343	88.0

#### Analysis of gene expression levels and screening of differentially expressed genes

**Fig. 10:**
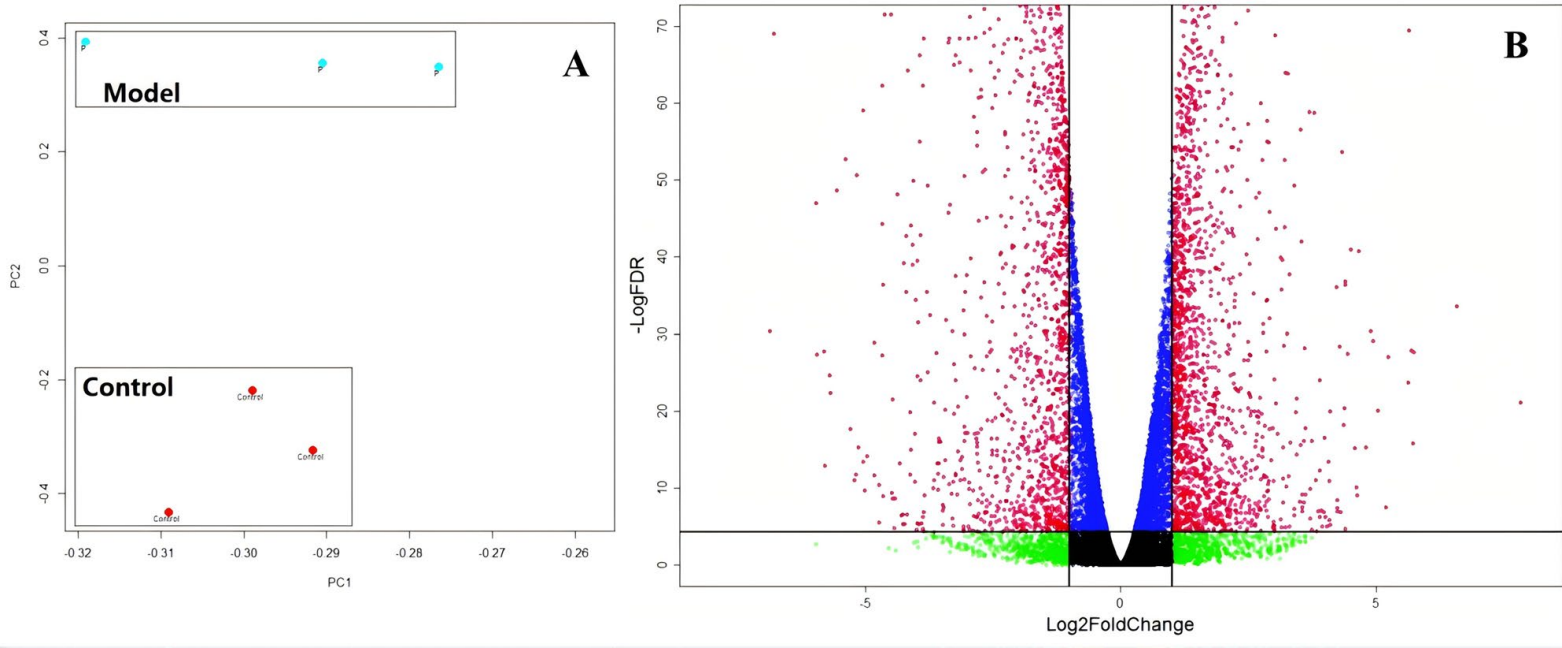
PCA and volcano plot of transcriptomic differences between control and PCS-treated groups. (**A**) PCA plot sho clear separation between control and PCS-treated samples, indicating distinct gene expression profiles. (**B**) Volcano plot displays differential gene expression; red dots represent significantly upregulated (fold change > 2) and downregulated (fold change < 0.5) genes with FDR < 0.05

In this study, gene expression levels were quantified using the RPKM method for visualization purposes. For differential expression analysis, raw counts were normalized using the TMM method. The normalized data obtained after correction served as the basis for identifying differentially expressed genes. PCA was employed to characterize the clustering patterns between the control group and the PCS-treated group. As illustrated in Fig. 10A, a clear separation trend between the two groups is evident, suggesting that PCS stimulation induces substantial alterations in the osteoblast transcriptome. Figure 10B presents a volcano plot, constructed using the FDR values and fold change values derived from the t-test analysis of the two sample groups. This plot highlights significant differences in the transcriptome profiles between the groups. In the volcano plot, variables with a fold change greater than 2 or less than 0.5, and an FDR value below 0.05 (highlighted in red), were classified as differentially expressed genes. A total of 3,679 significantly differentially expressed genes were identified between the control and PCS-treated groups, comprising 1,860 upregulated genes and 1,819 downregulated genes.

#### GO analysis and pathway analysis

**Fig. 11:**
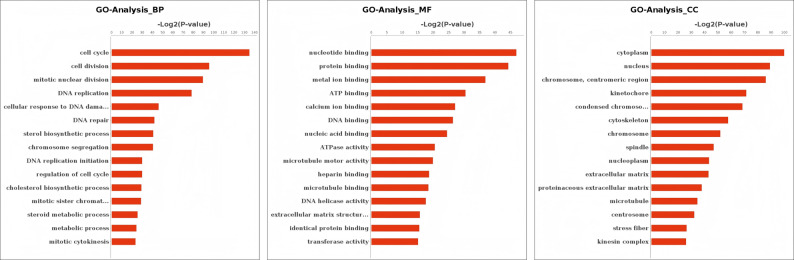
GO enrichment analysis of differentially expressed genes after PCS stimulation. Bar charts show the top 15 significantly enriched biological processes, molecular functions, and cellular components affected in osteoblasts

The 3,679 differentially expressed genes obtained through screening were subjected to significant analysis of gene function (GO analysis). Complete information annotation and source marking of GO were conducted through NCBI, Swissprot/Uniprot, The Gene Ontology, and AmiGo. The target genes were further classified according to GO, and the classification results were analyzed based on discrete distribution significance, false discovery rate, and enrichment degree. The gene function classification of the target genes with significant association, low false discovery rate was obtained. The results indicated that the genes altered in osteoblasts after PCS stimulation significantly affected 659 biological processes (biological process), 234 molecular functions (Molecular Function), and 150 cellular components (cellular component). Figure 11 presents the top 15 biological processes, molecular functions, and cellular components significantly altered in osteoblasts after PCS stimulation: the significantly altered biological processes include cell cycle, cell differentiation, mitotic nuclear division, DNA replication, cell response to DNA damage stimulus, DNA repair, steroid biosynthesis process, chromosome segregation, DNA replication initiation, regulation of cell cycle, cholesterol biosynthesis process, mitotic sister chromatid separation, steroid metabolism process, metabolic process, and mitotic cell division; the significantly altered molecular functions include nucleotide binding, protein binding, metal ion binding, ATP binding, calcium ion binding, DNA binding, nucleic acid binding, ATPase activity, microtubule motor activity, heparin binding, microtubule binding, DNA helicase activity, extracellular matrix structural constituent, identical protein binding, and transferase activity; the significantly altered cellular components include cytoplasm, nucleus, chromosome, centromere region, kinetochore, chromatin, cytoskeleton, chromosome, spindle, nucleoplasm, extracellular matrix, extracellular matrix protein, microtubule, centrosome, stress fiber, and kinesin complex.

**Fig. 12:**
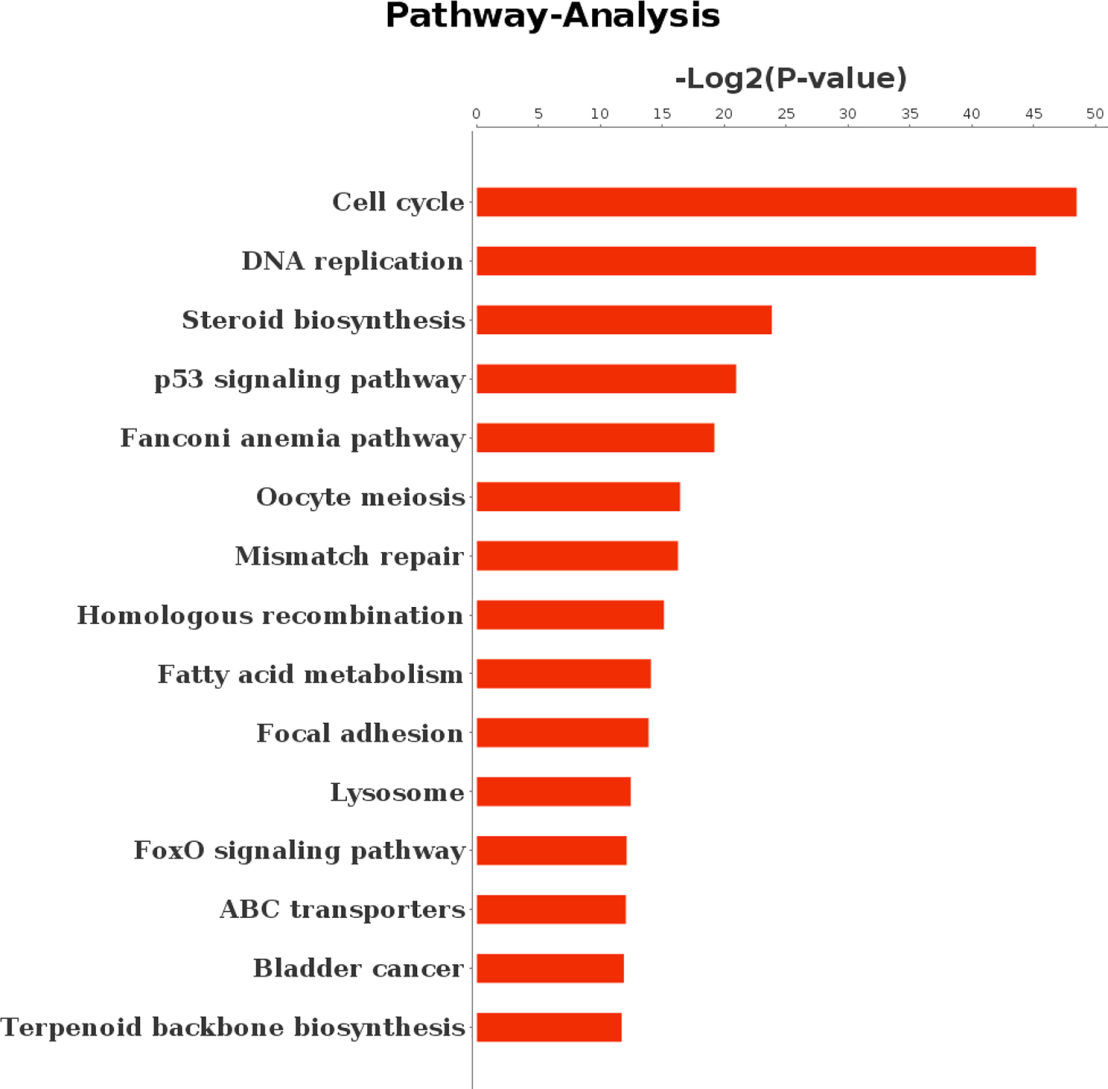
KEGG pathway enrichment analysis of differentially expressed genes after PCS stimulation. Bar chart displays the top 15 significantly enriched signaling pathways

Meanwhile, the significant analysis of signal transduction pathways (Pathway analysis) was conducted on the differentially expressed genes. The latest databases such as NCBI, KEGG, Biocarta, and Reactome were integrated to provide as complete information annotation as possible for pathways. The differentially expressed genes were classified according to KEGG pathways, and the genes were analyzed based on discrete distribution significance to obtain significant pathway classifications. The results indicated that the genes altered in osteoblasts after PCS stimulation affected 58 signaling pathways. Figure 12 presents the top 15 significantly altered signaling pathways in osteoblasts after PCS stimulation, including cell cycle, DNA replication, steroid biosynthesis, p53 signaling pathway, Fanconi anemia pathway, oocyte meiosis, mismatch repair, homologous recombination, fatty acid metabolism, focal adhesion, lysosome, FoxO signaling pathway, ABC transporter, bladder cancer, and terpenoid backbone biosynthesis. Table S3 lists all 58 signaling pathways significantly associated with PCS stimulation.

### Comparative analysis of metabolic pathways and related biological functions based on transcriptome and metabolome data

**Fig. 13:**
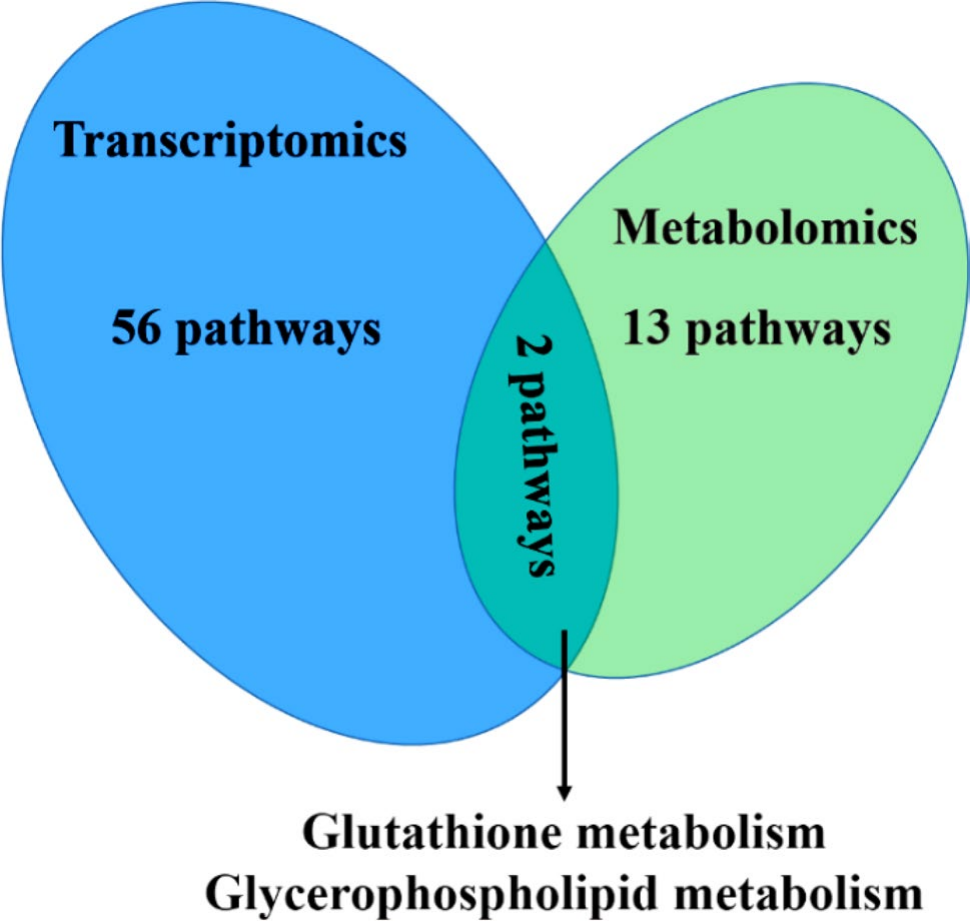
Integrative transcriptomic and metabolomic analysis identified glutathione metabolism and glycerophospholipid metabolism as significantly altered pathways in PCS-treated cells

At the downstream functional response molecule level, based on the differential metabolites screened by metabolomics and the intelligent pathway analysis on the MetPA platform, we identified a total of 15 significantly altered metabolic pathways at the metabolic level. To trace the upstream regulatory genes of the metabolic group differences, we used transcriptome sequencing to screen for differentially expressed genes in osteoblasts after PCS stimulation and identified a total of 58 significantly altered metabolic pathways at the transcriptional level through gene enrichment analysis. By comparing the significantly altered metabolic pathways at the metabolic and transcriptional levels, we ultimately found that the glutathione metabolism and glycerophospholipid metabolism in the cells of the control group and the PCS treatment group showed significant differences at both the metabolic and transcriptional levels, as shown in Fig. 13.

We further analyzed the significant differences in glutathione metabolism at the metabolic and transcriptional levels. Compared with the blank control group, the osteoblasts after PCS treatment had significantly increased ornithine levels and significantly decreased glutathione, glutamic acid, and spermidine; while the differentially expressed genes and their encoded enzymes at the transcriptional level included glutathione synthetase (Gss), γ-glutamyl transpeptidase 1 (Ggt1), ribonucleoside-diphosphate reductase subunit M2 (Rrm2), ornithine decarboxylase (Odc1), isocitrate dehydrogenase 1 (Idh1), 5-oxoprolinase (Oplah), γ-glutamylcyclotransferase (Ggct), aminopeptidase N (Anpep), microsomal glutathione S-transferase 3 (Mgst3), among which the upregulated genes included Ggt1, Oplah, Ggct, Gss, Anpep, and Odc1, and the downregulated genes included Rrm2, Idh1, and Mgst3.

Similarly, we analyzed the significant differences in glycerophospholipid metabolism at the metabolic and transcriptional levels. Compared with the blank control group, the osteoblasts after PCS treatment had significantly increased glycerophosphocholine and significantly decreased LysoPC (P-16:0); while the differentially expressed genes at the transcriptional level included lysophosphatidylcholine acyltransferase 3 (Lpcat3), phosphatidylserine synthase 2 (Ptdss2), phosphatidylethanolamine cytidylyltransferase (Pcyt2), choline phosphocytidyltransferase A (Pcyt1a), membrane-associated phospholipase B1 (Plb1), lipin 1 (Lpin1), membrane-bound O-acyltransferase 1 (Mboat1), truncated diacylglycerol kinase αFlt1-Pr2 (Dgka), and tafazzin (Taz), among which the upregulated genes included Lpin1, Dgka, Taz, and Ptdss2, and the downregulated genes included Lpcat3, Pcyt2, Pcyt1a, Plb1, and Mboat1.

**Fig. 14:**
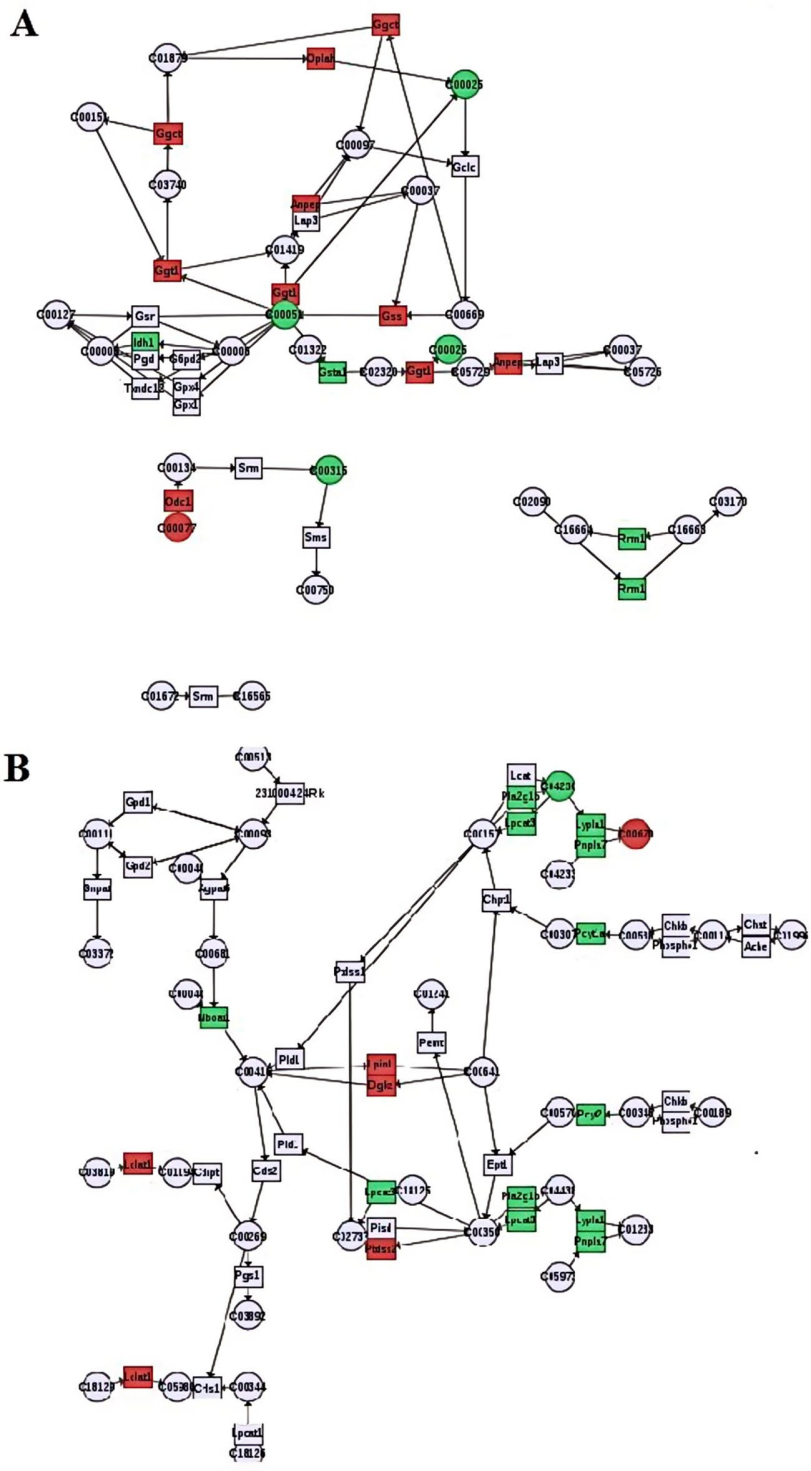
The Biochemical network of enriched pathway based on KEGG database: (**A**) Glutathione metabolism, (**B**) Glycerophospholipid metabolism. C00025: L-Glutamic acid, C00051: Glutathione, C00077: Ornithine, C00315: Spermidine, C04230: LysoPC(P-16:0), C00670: Glycerophosphocholine

**Fig. 15:**
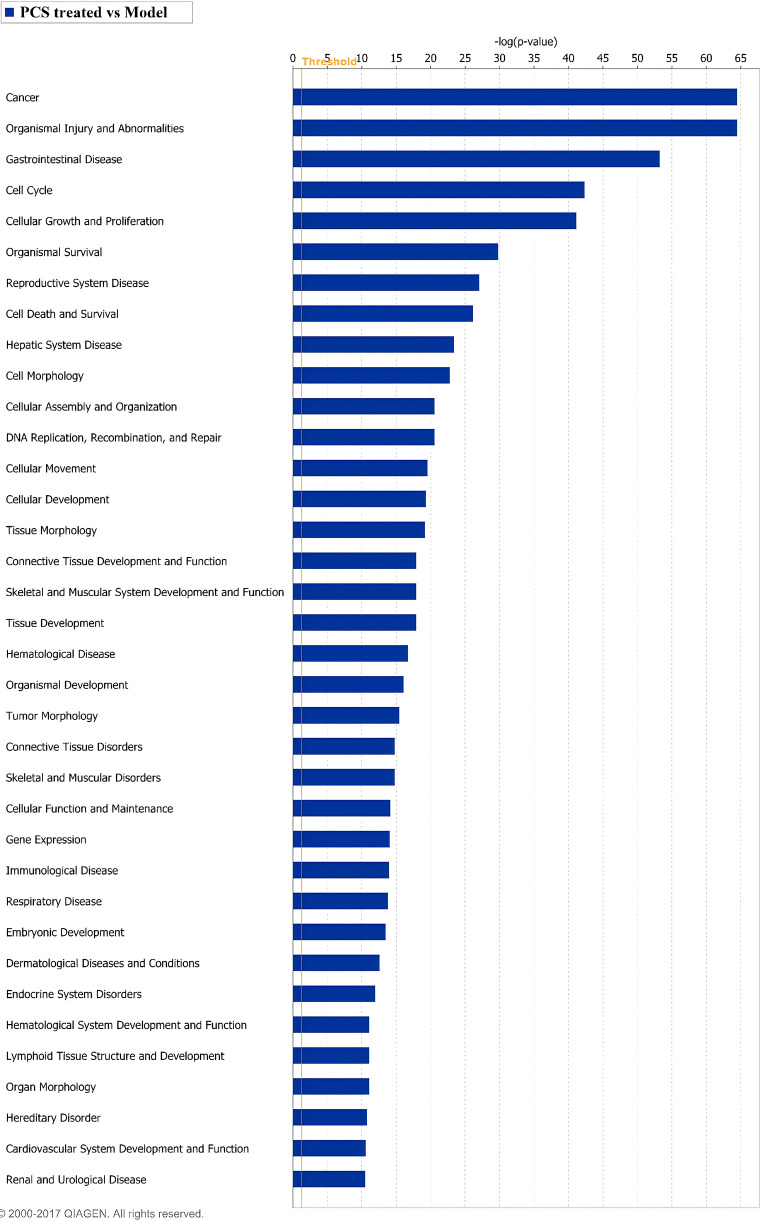
Functions significantly altered both metabolic and transcriptional level in PCS treated group by IPA

**Fig. 16:**
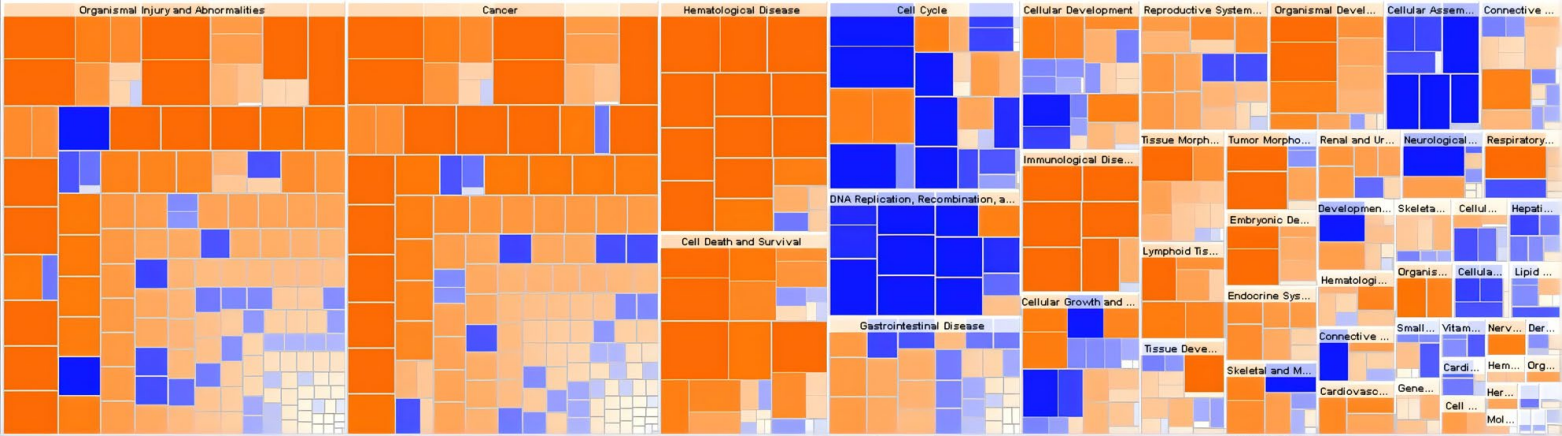
The categories of functions significantly altered both metabolic and transcriptional level in PCS treated group by IPA

Tables 3 and 4 list the differentially expressed genes and differential metabolites of glutathione metabolism and glycerophospholipid metabolism at the metabolic and transcriptional levels, and the specific enriched and focused glutathione metabolism and glycerophospholipid metabolism pathways were obtained based on the KEGG database, as shown in Fig. 14. In addition, based on the IPA analysis results of the imported differential metabolites and differentially expressed genes, we obtained the cell functions that were significantly dysregulated at both the transcriptional and metabolic levels and classified them. Among the upregulated biological function categories, there were 11 items, including Cancer, Organismal Injury and Abnormalities, Organismal Survival, Cell Death and Survival, Cancer, Hematological Disease, Organismal Injury and Abnormalities, Cancer, Hematological Disease, Immunological Disease, Organismal Injury and Abnormalities, Embryonic Development, Organismal Development, Tissue Development, Lymphoid Tissue Structure and Development, Tissue Morphology, Cancer, Organismal Injury and Abnormalities, Respiratory Disease, Cellular Growth and Proliferation, Lymphoid Tissue Structure and Development, Hematological Disease, Immunological Disease, Embryonic Development, Organismal Development, and Cancer, Cell Death and Survival, Organismal Injury and Abnormalities, Tumor Morphology. There are six down-regulated biological function categories, including Cell Cycle, Cellular Assembly and Organization, DNA Replication, Recombination, and Repair, DNA Replication, Recombination, and Repair, Cellular Assembly and Organization, DNA Replication, Recombination, and Repair, Cell Cycle, Cell Cycle, DNA Replication, Recombination, and Repair, and Neurological Disease, Organismal Injury and Abnormalities. The specific significantly changed functions are shown in Fig. 15, and the classification items are shown in Fig. 16.

**Table 3:** Gene variables in Glutathione metabolism and Glycerophospholipid metabolism

Pathway term	QueryID	Description	*p* value	FDR
Glutathione metabolism	Gss	Glutathione synthetase	0.02	0.12
	Ggt1	Gamma-glutamyltranspeptidase 1	0.02	0.12
	Rrm2	Ribonucleoside-diphosphate reductase subunit M2	0.02	0.12
	Odc1	Ornithine decarboxylase	0.02	0.12
	Idh1	Isocitrate dehydrogenase [NADP] cytoplasmic	0.02	0.12
	Oplah	5-oxoprolinase	0.02	0.12
	Ggct	Gamma-glutamylcyclotransferase	0.02	0.12
	Anpep	Aminopeptidase N	0.02	0.12
	Mgst3	Microsomal glutathione S-transferase 3	0.02	0.12
Glycerophospholipid metabolism	Lpcat3	Lysophosphatidylcholine acyltransferase 3	0.04	0.21
	Ptdss2	Phosphatidylserine synthase 2	0.04	0.21
	Pcyt2	Phosphate cytidylyltransferase 2, ethanolamine, isoform CRA_b	0.04	0.21
	Pcyt1a	Choline-phosphate cytidylyltransferase A	0.04	0.21
	Plb1	Phospholipase B1, membrane-associated	0.04	0.21
	Lpin1	Phosphatidate phosphatase LPIN1	0.04	0.21
	Mboat1	Membrane bound O-acyltransferase domain containing 1	0.04	0.21
	Dgka	Truncated diacylglycerol kinase alpha Flt1-Pr2	0.04	0.21
	Taz	Tafazzin	0.04	0.21

**Table 4:** Metabolites variables in Glutathione metabolism and Glycerophospholipid metabolism

Pathway term	Metabolites	KEGG ID	*p* value	VIP	FC^a^
Glutathione metabolism	Glutathione	C00051	< 0.05	2.03	0.64
	L-Glutamic acid	C00025	< 0.01	2.08	0.53
	Ornithine	C00077	< 0.001	3.29	1.62
	Spermidine	C00315	< 0.01	1.09	0.44
Glycerophospholipid metabolism	Glycerophosphocholine	C00670	< 0.01	3.94	4.12
	LysoPC(P-16:0)	C04230	< 0.01	1.73	0.37

^a^: PCS treated group compared with control group

## Discussion

In this study, we employed a series of bioinformatics approaches to investigate the potential cytotoxic effects of PCS on osteoblasts. Initially, we conducted metabolic profiling using UHPLC-QTOF/MS and transcriptional analysis through RNA-seq. By leveraging the MetPA platform, we delved deeply into the significantly altered metabolic pathways. By combining metabolomics and transcriptomics data, we focused on pathways that exhibited significant changes at both the metabolic and transcriptional levels. Additionally, we utilized the IPA software to perform disease and functional enrichment analyses on the differential metabolites and expressed genes, adhering to the criteria of Fisher’s Exact Test (*p* < 0.05) and a Z-score (absolute value greater than 2) to identify crucial cellular biological functions. Ultimately, we identified two metabolic pathways, glutathione metabolism and glycerophospholipid metabolism, which showed significant differences at both the metabolic and transcriptional levels. Concurrently, we observed significant dysregulations related to cellular apoptosis, cell death, cell cycle, and DNA replication, recombination, and repair functions, consistent with preliminary results from cell viability and cell cycle assays. These results provide mechanistic insights into how uremic toxins may contribute to the pathogenesis of CKD-MBD.

CKD-MBD is a prevalent complication in patients with chronic kidney disease, characterized by imbalances in calcium, phosphorus, vitamin D, and parathyroid hormone levels, which contribute to bone lesions and vascular calcification [28, 29]. Fibroblast growth factor 23 (FGF23), klotho protein, and the calcium-sensing receptor play critical roles in maintaining mineral homeostasis [30–33]. Despite recent advances in understanding this condition, treating CKD-induced bone metabolic disorders remains challenging due to the complexity of the underlying mechanisms. Specifically, these challenges stem from the intricate interplay of multiple factors leading to metabolic abnormalities, as well as the paucity of highly targeted and minimally invasive therapeutic options [34]. In our study, a comparative multi-omics approach combining metabolomics and transcriptomics revealed that PCS exerts its cytotoxic effects on osteoblasts by significantly disrupting glutathione metabolism and glycerophospholipid metabolism pathways.

Before discussing the mechanistic findings, it is important to address the experimental context. The PCS concentration (500 µg/mL) used in this in vitro study is higher than typical serum levels in CKD patients. This concentration was selected to model the plausible scenario of local tissue accumulation, given the protein-binding nature and poor clearance of PCS. This allows us to delineate the direct pathological potential of the toxin on osteoblasts in a controlled setting. Furthermore, our model utilizes the free form of PCS, which, while enabling the dissection of fundamental toxicological mechanisms, differs from the predominantly albumin-bound state in vivo. We acknowledge these as intentional simplifications to establish a clear mechanistic baseline; future studies employing more physiologically complex models are essential for translational validation.

### Significant changes in glutathione metabolism in osteoblasts induced by PCS

In our study, we observed significant alterations in glutathione metabolism in osteoblasts following PCS treatment, both at the metabolic and transcriptional levels, particularly notable were the downregulation of glutathione and glutamate. Glutathione, a tripeptide synthesized from glutamate, cysteine, and glycine through two ATP-consuming enzymatic reactions, is the most abundant low-molecular-weight thiol-containing compound within cells. It plays a crucial role in maintaining cellular redox balance through its cyclic conversion between reduced (GSH) and oxidized (GSSG) states [35]. Under normal physiological conditions, the production and clearance of ROS such as superoxide radicals, H2O2, and organic hydroperoxides, as well as reactive nitrogen species, are balanced, activating the glutathione antioxidant defense system. However, external stimuli like PCS treatment can increase the production of intracellular ROS and superoxide radicals beyond the antioxidative capacity of the cell, leading to rapid glutathione depletion and oxidative stress [36, 37]. This oxidative stress not only continues to deplete glutathione but may also hinder the reduction of oxidized GSSG back to GSH, potentially causing further oxidative damage to membrane proteins, lipids, and nucleic acids, and ultimately leading to cell apoptosis or senescence [38].

Additionally, at the transcriptional level, our study highlighted a significant activation of the γ-Glutamyl Cycle, with notable upregulation in genes encoding glutathione synthesizing enzymes such as glutathione synthetase (Gss), γ-glutamyltransferase 1 (Ggt1), 5-oxoprolinase (Oplah), and γ-glutamylcyclotransferase (Ggct). The increased activity of these enzymes suggests that cells are attempting to enhance glutathione synthesis and recycling to combat oxidative stress [39, 40]. Especially, Ggt1 plays a pivotal role in maintaining intracellular glutathione levels and is currently the only known enzyme capable of hydrolyzing the γ-peptide bond between glutamate and glycine in glutathione, suggesting its activation as a response mechanism to oxidative stress [41].

Furthermore, significant changes in other genes were observed, such as the upregulation of aminopeptidase N (Anpep) and ornithine decarboxylase (Odc1), along with the downregulation of isocitrate dehydrogenase 1 (Idh1), microsomal glutathione S-transferase 3 (Mgst3), and ribonucleotide reductase M2 subunit (Rrm2). These changes reflect cellular adjustments in metabolic and defensive mechanisms in response to increased oxidative stress following PCS treatment [42–47]. This study not only reveals the potential cytotoxic effects of PCS on osteoblasts but also deepens our understanding of how cells respond to and adapt to oxidative stress. Further investigation into the specific mechanisms of these changes will be crucial for developing new therapeutic strategies.

### Significant changes in glycerophospholipid metabolism in osteoblasts induced by PCS

In this study, we observed that PCS treatment significantly impacts glycerophospholipid metabolism in osteoblasts, with notable alterations at both the metabolic and transcriptional levels. Metabolically, there was an upregulation of glycerophosphocholine (GPC) and a downregulation of lysophosphatidylcholine (LysoPC), suggesting potential disruption in the deacylation process of phosphatidylcholine (PC) [48, 49]. Transcriptomic analysis revealed expression changes in several key enzyme genes involved in this pathway, including lysophosphatidylcholine acyltransferase 3 (Lpcat3), phosphatidylethanolamine cytidylyltransferase (Pcyt2), choline phosphate cytidylyltransferase A (Pcyt1a), membrane-associated phospholipase B1 (Plb1), membrane-bound O-acyltransferase 1 (Mboat1), diacylglycerol kinase (Dgka), Tafazzin (Taz), phosphatidylserine synthase 2 (Ptdss2), and phosphatidic acid phosphatase LPIN1 (Lpin1).

Glycerophospholipids are crucial components of the cellular membrane bilayer, playing vital roles in cell signaling processes [50]. Plb1, which exhibits both lysophospholipase hydrolase and acyltransferase activities, is critical in maintaining cell membrane integrity and modulating immune responses [51, 52]. The downregulation of Plb1 following PCS treatment suggests that abnormalities in membrane phospholipid metabolism could trigger immune response dysfunctions. Lpcat3, pivotal in modulating cellular inflammatory responses by integrating arachidonic acid into phospholipids, exhibited reduced expression which could lead to membrane structure damage, thereby limiting cellular extension and proliferation, and potentially enhancing cell apoptosis [53]. Mboat1, a transmembrane protein, adjusts intracellular free cholesterol levels; its reduced expression may indicate that PCS treatment exacerbates cellular damage by inducing endoplasmic reticulum stress [54]. Additionally, decreased activity of crucial enzymes in the CDP-choline and CDP-ethanolamine pathways, such as Pcyt1a and Pcyt2, not only impacts the structural and functional integrity of the cell membrane but also increases cellular sensitivity to the cytotoxic effects of free cholesterol, leading to cellular dysfunction and death [55].

Previous studies have established that protein-bound uremic toxins, particularly indoxyl sulfate (IS) and p-cresyl sulfate (PCS), exert detrimental effects on bone cells through multiple mechanisms. Specifically, PCS has been shown to inhibit osteoblast viability, induce apoptosis, and reduce parathyroid hormone (PTH)-induced cAMP production by activating the JNK and p38 MAPK signaling pathways [56, 57]. Additionally, both IS and PCS impair osteogenic differentiation of mesenchymal stem cells (MSCs) by triggering cellular senescence and downregulating key osteogenic markers such as collagen type I and alkaline phosphatase (ALP) activity [58]. Oxidative stress plays a central role in these processes, as uremic toxins increase reactive oxygen species (ROS) production, leading to enhanced osteoclastogenesis and suppressed osteoblast differentiation via activation of NF-κB and MAPK pathways, as well as disruption of the Wnt/β-catenin signaling axis [59].

In the context of this established framework, our study extends the current understanding by providing a comprehensive, multi-omics perspective on PCS-induced osteoblast dysfunction. While previous work has focused on discrete signaling events (e.g., JNK/p38 activation) [56, 57], our combined metabolomic and transcriptomic analysis reveals that PCS broadly disrupts glutathione and glycerophospholipid metabolism—two pathways central to redox homeostasis and membrane integrity. These findings suggest that the previously reported oxidative stress [59] and MAPK activation [56, 57] may be downstream consequences of a more fundamental metabolic reprogramming. Importantly, our data identify specific metabolic nodes (e.g., depleted glutathione, altered lysophosphatidylcholine species) that have not been previously linked to PCS toxicity in osteoblasts. Thus, while our results align with prior observations of PCS-induced cytotoxicity and oxidative stress, they also uncover novel metabolic vulnerabilities that could serve as therapeutic targets. Moreover, the parallel transcriptomic changes we observed in genes governing glutathione synthesis (e.g., Gss) and phospholipid remodeling (e.g., Lpcat3) corroborate the metabolomic findings and provide a mechanistic link between PCS exposure and the disruption of osteoblast function at both the metabolic and transcriptional levels.

Taken together, our study not only reinforces the established paradigm of PCS-induced oxidative stress and MAPK-mediated toxicity but also introduces a new dimension—namely, the coordinated disruption of glutathione and glycerophospholipid metabolism—that may underlie the broader cellular dysfunction observed in uremic bone disease. Future studies should explore whether targeting these metabolic pathways (e.g., with glutathione precursors or lipid-modulating agents) can attenuate PCS-induced osteoblast injury, thereby offering novel therapeutic strategies for CKD-MBD.

### Limitations

This study is subject to several limitations. First, as noted, the in vitro model employs a high concentration of free PCS. While mechanistically informative, the findings require validation in models that better approximate the in vivo milieu, including physiological toxin concentrations and protein-binding dynamics. Second, this study focused on cytotoxicity and pathway analysis; it did not include functional assays of osteoblast differentiation or mineralization. The transcriptomic data suggest broad metabolic dysfunction that would likely impair these specialized functions, but direct experimental confirmation is a critical objective of our ongoing research. Third, the multivariate models (e.g., PLS-DA) used to identify key metabolic disruptions were assessed for robustness using 200 permutation tests. While this internal validation helped mitigate overfitting concerns, the models await external validation with an independent sample set to fully confirm their generalizability. Additionally, multiple-testing correction (e.g., FDR) was not applied to the univariate statistical analyses, which may increase the risk of false positives; future studies incorporating such corrections are needed to further validate these findings. Fourth, metabolite identification was based on accurate mass matching without MS/MS confirmation, corresponding to MSI Level 3. Future studies incorporating MS/MS validation are needed to confirm the identities of these metabolites. Finally, differential expression analysis in this study was performed using Student’s t-test with FDR correction (Benjamini-Hochberg). While this approach is commonly used for exploratory analysis, we acknowledge that more specialized RNA-seq frameworks (e.g., DESeq2, edgeR) are better suited for modeling the discrete nature of count data and may offer greater statistical robustness. Future validation studies using these methods would help confirm the reliability of the transcriptomic findings. The causal relationships within the identified metabolic networks need further elucidation through targeted biochemical and genetic interventions.

## Conclusions

This study employed a parallel metabolomic and transcriptomic approach to investigate the cytotoxic mechanisms of PCS, a representative protein-bound uremic toxin, in MC3T3-E1 osteoblasts. Our analysis revealed widespread metabolic and transcriptional perturbations induced by PCS, with a notable convergence on the glutathione and glycerophospholipid metabolism pathways. These molecular changes were functionally linked to impaired cellular viability, increased apoptosis, and cell cycle arrest. The present work establishes a foundational framework for understanding how uremic toxins may contribute to bone metabolism disorders in CKD. Future studies are warranted to validate these findings in vivo and to delineate the precise causal relationships within the identified pathways.

## Electronic Supplementary Material

Below is the link to the electronic supplementary material.

Supplementary Material 1

## Data Availability

The multi-omics data generated in this study have been deposited in the OMIX repository. The transcriptomic data are available under accession number OMIX012838, and the metabolomic data under accession number OMIX012839.

## Abbreviations

CKD-MBD: Chronic kidney disease-mineral bone disorder
CKD: Chronic kidney disease
PCS: P-cresyl sulfate
UTs: Uremic toxins
UHPLC-QTOF/MS: Ultra-high performance liquid chromatography and quadrupole time-of-flight mass spectrometry
RNA-seq: RNA sequencing
IPA: Ingenuity Pathway Analysis
GSH: Reduced glutathione
GSSG: Oxidized glutathione
QC: Quality control
PI: Proliferation index
PCA: Principal component analysis
PLS-DA: Partial least squares-discriminant analysis
