# Supplementary material for: Parallel metabolomic and transcriptomic profiling reveals the cytotoxic mechanism of protein-bound uremic toxin p-cresol sulfate via disruption of glutathione and glycerophospholipid metabolism in MC3T3-E1 osteoblasts
Source: BMC Nephrol. 2026 Apr 28;27:369. doi: 10.1186/s12882-026-05004-x (PMC13270783; doi:10.1186/s12882-026-05004-x)
Supplement: Supplementary file 1 — Supplementary Material 1 [file 12882_2026_5004_MOESM1_ESM.docx]

**Supplementary Materials**

**Table S1** The identified significantly differential metabolites in intracellular extract

| **m/z** | **Metabolites** | **Formula** | **p value** | **VIP** | **FC^a^** | **MSI Level** |
| --- | --- | --- | --- | --- | --- | --- |
| 299.10 | 1-Methyladenosine | C11H15N5O4 | <0.001 | 1.41 | 0.29 | Level 3 |
| 298.10 | 1-Methylguanosine | C11H15N5O5 | <0.001 | 3.59 | 0.30 | Level 3 |
| 110.98 | 3-Furoic acid | C5H4O3 | <0.001 | 3.31 | 14.03 | Level 3 |
| 196.10 | 3-Hydroxyhippuric acid | C9H9NO4 | <0.001 | 1.86 | 4.95 | Level 3 |
| 273.17 | 3-Hydroxymelatonin | C13H18N2O3 | <0.05 | 1.31 | 3.10 | Level 3 |
| 130.06 | 3-Methylindole | C9H9N | <0.001 | 1.09 | 0.27 | Level 3 |
| 429.26 | 3-Oxocholic acid | C24H38O5 | <0.001 | 1.61 | 0.21 | Level 3 |
| 176.04 | 4-Hydroxyproline | C5H9NO3 | <0.01 | 1.24 | 0.40 | Level 3 |
| 190.02 | 5-Hydroxyindoleacetic acid | C10H9NO3 | <0.05 | 1.17 | 1.63 | Level 3 |
| 203.05 | 5-Hydroxykynurenamine | C9H12N2O2 | <0.001 | 1.32 | 5.27 | Level 3 |
| 488.18 | 7-hydroxymethotrexate | C20H22N8O6 | <0.001 | 1.87 | 8.12 | Level 3 |
| 401.35 | 7-Ketocholesterol | C27H44O2 | <0.001 | 4.09 | 3.10 | Level 3 |
| 407.28 | 7-Ketodeoxycholic acid | C24H38O5 | <0.01 | 1.23 | 0.23 | Level 3 |
| 343.09 | 7-Methylguanosine | C11H16N5O5 | <0.001 | 1.49 | 0.63 | Level 3 |
| 277.03 | Acetyl citrate | C8H8O8 | <0.01 | 1.37 | 2.87 | Level 3 |
| 268.13 | Adenosine | C10H13N5O4 | <0.001 | 1.18 | 4.12 | Level 3 |
| 249.08 | Aspartyl-Aspartate | C8H12N2O7 | <0.001 | 1.48 | 8.06 | Level 3 |
| 261.01 | Caffeic acid 3-sulfate | C9H8O7S | <0.001 | 1.11 | 2.54 | Level 3 |
| 426.36 | Cholic acid | C24H40O5 | <0.01 | 1.96 | 1.83 | Level 3 |
| 191.02 | Citric acid | C5H9NO4 | <0.05 | 2.01 | 2.81 | Level 3 |
| 130.06 | Creatine | C4H9N3O2 | <0.01 | 3.74 | 0.53 | Level 3 |
| 316.32 | Dehydrophytosphingosine | C18H37NO3 | <0.001 | 1.00 | 2.09 | Level 3 |
| 328.24 | Dihydroceramide | C19H39NO3 | <0.001 | 1.07 | 3.86 | Level 3 |
| 119.09 | Ethyl lactate | C5H10O3 | <0.001 | 2.09 | 1.85 | Level 3 |
| 486.16 | Folic acid | C19H19N7O6 | <0.001 | 2.13 | 6.94 | Level 3 |
| 306.08 | Glutathione | C10H17N3O6S | <0.05 | 2.03 | 0.64 | Level 3 |
| 258.11 | Glycerophosphocholine | C8H20NO6P | <0.01 | 3.94 | 4.12 | Level 3 |
| 288.29 | Heptadecanoic acid | C17H34O2 | <0.001 | 1.82 | 2.88 | Level 3 |
| 129.06 | Heptanoic acid | C7H14O2 | <0.01 | 3.77 | 0.47 | Level 3 |
| 158.04 | Homocysteine | C4H9NO2S | <0.001 | 2.96 | 12.63 | Level 3 |
| 183.08 | Hydroxyphenyllactic acid | C9H10O4 | <0.001 | 1.05 | 1.78 | Level 3 |
| 137.01 | Hypoxanthine | C5H4N4O | <0.001 | 5.79 | 19.72 | Level 3 |
| 308.12 | Inodxyl glucuronide | C14H15NO7 | <0.01 | 3.21 | 0.65 | Level 3 |
| 133.11 | L-Asparagine | C4H8N2O3 | <0.001 | 1.91 | 1.76 | Level 3 |
| 162.11 | L-Carnitine | C7H15NO3 | <0.001 | 2.55 | 0.32 | Level 3 |
| 272.19 | L-Furosine | C12H18N2O4 | <0.05 | 1.51 | 1.77 | Level 3 |
| 203.05 | L-Galactose | C6H12O6 | <0.001 | 2.09 | 1.99 | Level 3 |
| 146.05 | L-Glutamic acid | C5H9NO4 | <0.01 | 2.08 | 0.53 | Level 3 |
| 173.15 | L-Histidine | C6H9N3O2 | <0.05 | 1.10 | 1.82 | Level 3 |
| 132.10 | L-Isoleucine | C6H13NO2 | <0.001 | 7.74 | 2.05 | Level 3 |
| 400.34 | L-Palmitoylcarnitine | C23H45NO4 | <0.001 | 2.09 | 3.09 | Level 3 |
| 166.09 | L-Phenylalanine | C9H11NO2 | <0.01 | 4.88 | 1.56 | Level 3 |
| 182.08 | L-Tyrosine | C9H11NO3 | <0.001 | 2.58 | 1.82 | Level 3 |
| 118.09 | L-Valine | C5H11NO2 | <0.001 | 8.34 | 1.96 | Level 3 |
| 480.31 | LysoPC(15:0) | C23H48NO7P | <0.01 | 1.70 | 0.55 | Level 3 |
| 538.32 | LysoPC(16:1(9Z)) | C24H48NO7P | <0.05 | 1.32 | 0.65 | Level 3 |
| 544.34 | LysoPC(20:4(5Z,8Z,11Z,14Z)) | C28H50NO7P | <0.05 | 2.43 | 1.69 | Level 3 |
| 478.29 | LysoPC(P-16:0) | C24H50NO6P | <0.01 | 1.73 | 0.37 | Level 3 |
| 545.34 | LysoPE(0:0/22:5(4Z,7Z,10Z,13Z,16Z)) | C27H46NO7P | <0.05 | 1.37 | 1.70 | Level 3 |
| 440.31 | LysoPE(15:0/0:0) | C20H42NO7P | <0.001 | 1.20 | 4.05 | Level 3 |
| 452.28 | LysoPE(16:0/0:0) | C21H44NO7P | <0.001 | 1.60 | 0.37 | Level 3 |
| 157.04 | Malic acid | C4H6O5 | <0.001 | 12.60 | 24.37 | Level 3 |
| 284.09 | N4-Acetylcytidine | C11H15N3O6 | <0.001 | 3.22 | 7.39 | Level 3 |
| 215.13 | N-Acetylhistidine | C8H11N3O3 | <0.05 | 1.12 | 2.42 | Level 3 |
| 125.93 | Nitrosylsulfuric acid | HNO5S | <0.05 | 2.65 | 0.41 | Level 3 |
| 307.11 | Nopalinic acid | C10H18N2O6 | <0.01 | 8.10 | 0.64 | Level 3 |
| 327.23 | Oleic acid | C18H34O2 | <0.001 | 2.18 | 4.05 | Level 3 |
| 133.11 | Ornithine | C5H12N2O2 | <0.001 | 3.29 | 1.62 | Level 3 |
| 179.02 | Orotic acid | C5H4N2O4 | <0.001 | 2.27 | 5.17 | Level 3 |
| 274.27 | Palmitic acid | C16H32O2 | <0.001 | 1.60 | 2.48 | Level 3 |
| 218.10 | Pantothenic acid | C9H17NO5 | <0.05 | 1.38 | 0.61 | Level 3 |
| 283.08 | p-Cresol glucuronide | C13H16O7 | <0.001 | 8.67 | 7.84 | Level 3 |
| 187.01 | p-Cresol sulfate | C7H8O4S | <0.001 | 7.02 | 47.49 | Level 3 |
| 192.03 | Phenol sulphate | C6H6O4S | <0.001 | 2.23 | 1.80 | Level 3 |
| 138.05 | Purine | C5H4N4 | <0.001 | 2.48 | 15.09 | Level 3 |
| 124.99 | Pyrimidine | C4H4N2 | <0.001 | 3.54 | 23.23 | Level 3 |
| 146.12 | Spermidine | C7H19N3 | <0.01 | 1.09 | 0.44 | Level 3 |
| 302.31 | Sphinganine | C18H39NO2 | <0.05 | 1.11 | 0.36 | Level 3 |
| 249.21 | Sterol | C17H28O | <0.05 | 3.69 | 1.80 | Level 3 |
| 117.02 | Succinic acid | C4H6O4 | <0.05 | 1.08 | 1.85 | Level 3 |
| 124.01 | Taurine | C2H7NO3S | <0.05 | 1.02 | 0.64 | Level 3 |
| 176.01 | Thiocysteine | C3H7NO2S2 | <0.001 | 3.06 | 10.56 | Level 3 |
| 309.11 | Valyl-Phenylalanine | C14H20N2O3 | <0.01 | 1.81 | 0.65 | Level 3 |
| 271.00 | Vanillic acid 4-sulfate | C8H8O7S | <0.05 | 2.52 | 2.09 | Level 3 |

^a^: PCS treated group compared with control group

**Table S2** QC results for transcriptome sequencing

| Sample | Encoding | TotalReads_Before | TotalReads_After | ReadsFilter% |
| --- | --- | --- | --- | --- |
| Control1 | IonProton | 17522744 | 16889710 | 96.39 |
| Control2 | IonProton | 14619737 | 14066769 | 96.22 |
| Control3 | IonProton | 17401355 | 16599434 | 95.39 |
| Model1 | IonProton | 16986752 | 16159659 | 95.13 |
| Model2 | IonProton | 16981745 | 16255976 | 95.73 |
| Model3 | IonProton | 16448457 | 15711523 | 95.52 |

**Table S3** Summary of significantly changed pathways

| **PathwayID** | **PathwayTerm** | **p Value** | **FDR** | **Enrichment** |
| --- | --- | --- | --- | --- |
| PATH:04110 | Cell cycle | 2.597E-15 | 7.116E-13 | 3.078043601 |
| PATH:03030 | DNA replication | 2.527E-14 | 3.462E-12 | 5.081675757 |
| PATH:00100 | Steroid biosynthesis | 6.599E-08 | 6.027E-06 | 5.006787903 |
| PATH:04115 | p53 signaling pathway | 4.867E-07 | 3.334E-05 | 2.757361454 |
| PATH:03460 | Fanconi anemia pathway | 1.641E-06 | 8.994E-05 | 2.955189902 |
| PATH:04114 | Oocyte meiosis | 1.116E-05 | 0.0004928 | 2.137002053 |
| PATH:03430 | Mismatch repair | 1.259E-05 | 0.0004928 | 3.81788509 |
| PATH:03440 | Homologous recombination | 2.747E-05 | 0.000941 | 3.39746322 |
| PATH:01212 | Fatty acid metabolism | 5.719E-05 | 0.0017412 | 2.634340712 |
| PATH:04510 | Focal adhesion | 6.543E-05 | 0.0017926 | 1.723860776 |
| PATH:04142 | Lysosome | 0.0001778 | 0.0044289 | 1.903769259 |
| PATH:04068 | FoxO signaling pathway | 0.0002224 | 0.0049108 | 1.842954408 |
| PATH:02010 | ABC transporters | 0.000233 | 0.0049108 | 2.545256727 |
| PATH:05219 | Bladder cancer | 0.0002607 | 0.0051019 | 2.613433246 |
| PATH:00900 | Terpenoid backbone biosynthesis | 0.0002952 | 0.0053915 | 3.326187768 |
| PATH:05200 | Pathways in cancer | 0.0006528 | 0.0111791 | 1.467971015 |
| PATH:01040 | Biosynthesis of unsaturated fatty acids | 0.0010123 | 0.0163166 | 2.927045236 |
| PATH:03410 | Base excision repair | 0.0013958 | 0.0212468 | 2.439204363 |
| PATH:04512 | ECM-receptor interaction | 0.0017977 | 0.0249438 | 1.870056678 |
| PATH:04710 | Circadian rhythm | 0.0018207 | 0.0249438 | 2.596572387 |
| PATH:03420 | Nucleotide excision repair | 0.0019622 | 0.0256022 | 2.276590739 |
| PATH:04151 | PI3K-Akt signaling pathway | 0.0022932 | 0.0285606 | 1.396809336 |
| PATH:05222 | Small cell lung cancer | 0.003498 | 0.0416721 | 1.808848179 |
| PATH:00061 | Fatty acid biosynthesis | 0.0041353 | 0.0459105 | 4.878408726 |
| PATH:04722 | Neurotrophin signaling pathway | 0.0041889 | 0.0459105 | 1.626136242 |
| PATH:04540 | Gap junction | 0.0066004 | 0.0687997 | 1.746248578 |
| PATH:05034 | Alcoholism | 0.0069039 | 0.0687997 | 1.470877003 |
| PATH:00511 | Other glycan degradation | 0.0070306 | 0.0687997 | 2.845738424 |
| PATH:05215 | Prostate cancer | 0.0075585 | 0.0705196 | 1.726627808 |
| PATH:00240 | Pyrimidine metabolism | 0.0077211 | 0.0705196 | 1.656818058 |
| PATH:04810 | Regulation of actin cytoskeleton | 0.0086687 | 0.0766202 | 1.43026074 |
| PATH:04390 | Hippo signaling pathway | 0.0090546 | 0.0774058 | 1.52054298 |
| PATH:05133 | Pertussis | 0.0093226 | 0.0774058 | 1.779959941 |
| PATH:04668 | TNF signaling pathway | 0.0109731 | 0.0884307 | 1.611217561 |
| PATH:00620 | Pyruvate metabolism | 0.0126383 | 0.0982345 | 1.995712661 |
| PATH:04360 | Axon guidance | 0.0130231 | 0.0982345 | 1.529053481 |
| PATH:05218 | Melanoma | 0.0132652 | 0.0982345 | 1.752104543 |
| PATH:00480 | Glutathione metabolism | 0.0161893 | 0.1167333 | 1.829403272 |
| PATH:05161 | Hepatitis B | 0.0207503 | 0.1457838 | 1.463522618 |
| PATH:04978 | Mineral absorption | 0.0212927 | 0.1458552 | 1.868326746 |
| PATH:04915 | Estrogen signaling pathway | 0.0222549 | 0.1482304 | 1.568059948 |
| PATH:04070 | Phosphatidylinositol signaling system | 0.0232624 | 0.1482304 | 1.626136242 |
| PATH:03018 | RNA degradation | 0.0232624 | 0.1482304 | 1.626136242 |
| PATH:04914 | Progesterone-mediated oocyte maturation | 0.0238933 | 0.1487904 | 1.59809941 |
| PATH:04066 | HIF-1 signaling pathway | 0.025143 | 0.153093 | 1.516262172 |
| PATH:01100 | Metabolic pathways | 0.0257045 | 0.1531093 | 1.13270178 |
| PATH:05203 | Viral carcinogenesis | 0.0263723 | 0.1537447 | 1.342094977 |
| PATH:00564 | Glycerophospholipid metabolism | 0.0366652 | 0.2058805 | 1.527853282 |
| PATH:00072 | Synthesis and degradation of ketone bodies | 0.036818 | 0.2058805 | 2.927045236 |
| PATH:04015 | Rap1 signaling pathway | 0.0391555 | 0.214572 | 1.321235697 |
| PATH:04270 | Vascular smooth muscle contraction | 0.0424754 | 0.2212725 | 1.418142072 |
| PATH:00603 | Glycosphingolipid biosynthesis - globo series | 0.0435329 | 0.2212725 | 2.439204363 |
| PATH:05216 | Thyroid cancer | 0.0436039 | 0.2212725 | 1.951363491 |
| PATH:01200 | Carbon metabolism | 0.0439762 | 0.2212725 | 1.450337729 |
| PATH:05213 | Endometrial cancer | 0.044416 | 0.2212725 | 1.688679944 |
| PATH:05205 | Proteoglycans in cancer | 0.0486926 | 0.2352768 | 1.295152759 |
| PATH:05162 | Measles | 0.0489444 | 0.2352768 | 1.38874409 |
| PATH:04611 | Platelet activation | 0.0498221 | 0.2353666 | 1.396491048 |
